# Transcriptomic profiling of wheat (*Triticum Aestivum* L.) response to infection by the wheat blast fungus *Magnaporthe Oryzae Triticum*

**DOI:** 10.3389/fpls.2026.1776686

**Published:** 2026-03-05

**Authors:** Xue Lyu, Chen Ji, Guanghao Guo, Xia Yan, He Zhao, Yu Wu

**Affiliations:** 1 Chengdu Institute of Biology, Chinese Academy of Sciences, Chengdu, China; 2 The Sainsbury Laboratory, University of East Anglia, Norwich Research Park, Norwich, United Kingdom; 3 University of Chinese Academy of Sciences, Beijing, China; 4 John Innes Centre, Department of Crop Genetics, Norwich Research Park, Norwich, United Kingdom; 5 Institute of Genetics and Developmental Biology, State Key Laboratory of Seed Innovation, Chinese Academy of Sciences, Beijing, China

**Keywords:** wheat blast, *Magnaporthe oryzae*, *Triticum aestivum* L., host susceptibility, effector candidates

## Abstract

**Introduction:**

The wheat blast fungus *Magnaporthe oryzae* pathotype *Triticum* (MoT) poses a severe threat to global wheat (*Triticum aestivum* L.) production, yet the molecular mechanisms underlying tissue invasion remain poorly understood.

**Methods:**

We performed dual RNA-seq analysis of MoT-inoculated wheat leaves at 0, 24, 36, and 48 hpi, mapping reads separately to the wheat and *M. oryzae* genomes to capture stage-specific host responses and pathogen gene expression across progressive infection stages.

**Results:**

Wheat exhibited pronounced stage-specific transcriptional reprogramming, with peak differential gene expression at 36 hpi and visible symptoms at 48 hpi. The 24 hpi stage was characterized by rapid induction of immune- and defense-related pathways, including innate immunity and detoxification processes, along with downregulation of cell wall and membrane biosynthesis. By 36 hpi, wheat maintained sustained activation of immune and detoxification pathways, while chloroplast- and photosynthesis-associated genes were broadly repressed, consistent with transcriptional features of metabolic constraint. At 48 hpi, coinciding with lesion initiation, transcriptomes showed persistent, metabolically costly immune and defense responses together with extensive suppression of photosynthesis- and chloroplast-associated functions, which were associated with metabolic strain and a transition toward necrosis. Analysis of pathogen-derived reads revealed temporal induction of multiple effector candidates, including known *M. oryzae* orthologs and additional effector-like proteins, highlighting coordinated temporal patterns between host immune and metabolic response as well as stage-specific pathogen effector expression.

**Discussion:**

Together, these findings provide a temporal framework for wheat blast susceptibility and highlight key host pathways and effector candidates that define critical windows for functional dissection of MoT virulence and wheat susceptibility.

## 1 Introduction

Wheat blast is a highly destructive fungal disease primarily affecting wheat spikes, causing bleaching and shriveling that lead to severe yield losses. First identified in Brazil in 1985, the disease has since spread across South America (Igarashi et al., 1986), with isolated outbreaks in the United States in 2011 (Farman et al., 2017), Asia in 2016–2017 (Islam et al., 2016), and Africa by 2018 (Tembo et al., 2020). Given its rapid spread, wheat blast poses a major threat to global wheat production and food security.

*Magnaporthe oryzae* (syn *Pyricularia oryzae*, *M. oryzae*) is a species complex responsible for blast disease in over 50 Poaceae species. Despite limited morphological differences, each pathotype exhibits host-specific pathogenicity, infecting major plants such as wheat, rice, barley, and crabgrass (Kato et al., 2000; Castroagudín et al., 2016). Wheat blast is caused by the *M. oryzae* pathotype *Triticum* (MoT), which primarily infects the base or upper rachis, disrupting spike formation and causing partial or complete spike bleaching and grain loss (Islam et al., 2016, 2019, 2020; Surovy et al., 2020; Gupta et al., 2021). MoT spreads via infected seeds, airborne conidia, and persists in crop residues, serving as inoculum for subsequent cycles (Pizolotto et al., 2019; Islam et al., 2020).

In addition to spike infection, necrotic leaf lesions are characteristic of wheat blast (Singh et al., 2021). Although wheat blast causes the greatest yield losses through spike infection, seedling leaves provide a controlled and reproducible system for resolving early host transcriptional responses. Leaf infections develop consistently under experimental conditions and permit precise temporal sampling, which is essential for transcriptome-based analyses (Latorre et al., 2023). Moreover, leaf inoculum has been positively associated with subsequent spike blast incidence, supporting the biological relevance of leaf-based models for investigating early host-pathogen dynamic (Chávez et al., 2023). Early infection processes on leaves, including appressorium formation and epidermal penetration, have been described in *Magnaporthe* species and provide a useful temporal reference sampling (Islam et al., 2016; Cruz and Valent, 2017). Following conidial inoculation, conidial germination and appressorium differentiation occur, with wax removal at appressorial sites reported between 6 and 24 hours post-infection (hpi). By approximately 48 hpi, the first visible symptoms appear as small, water-soaked lesions, marking the onset of lesion development. Highly susceptible cultivars may experience severe seedling infection, potentially resulting in complete plant death under favorable environmental conditions (Igarashi and Saunders, 1990; Singh, 2017).

Resistance to MoT is conferred by chromosomal segments and specific resistance genes. The 2NS chromosomal segment introgressed from *Aegilops ventricosa* into wheat chromosome 2A confers broad resistance, although resistance-breaking isolates have been reported (Singh et al., 2016; Ceresini et al., 2018; Juliana et al., 2020; He et al., 2022a). Resistance gene *Rmg7* and *Rmg8* confer complete resistance to MoT (Cruz et al., 2016; Anh et al., 2018), which can be suppressed by the effector *PWT4* but restored by Rwt4 (Inoue et al., 2021). Identification of effector candidates provides important context for interpreting host transcriptional responses and may support resistance research.

As a hemibiotrophic pathogen, MoT undergoes a transition from an initial biotrophic phase to a necrotrophic phase (Ceresini et al., 2018). This lifestyle is often associated with progressive reprogramming of host metabolism and defense responses (Parker et al., 2009). To investigate these molecular mechanisms, we performed transcriptomic analysis of MoT-inoculated wheat leaves. The susceptible spring wheat cultivar Cadenza was inoculated with MoT isolate BTJP, and infected leaf transcriptomes were sequenced at 0, 24, 36, and 48 hpi. These time points encompassed the transition from rapid host immune activation to a stage characterized by sustained defense engagement coinciding with the onset of visible lesion development. Transcriptome profiling across these time points enabled systematic characterization of host transcriptional dynamics, including temporal changes in immune-, detoxification-, and chloroplast-associated pathways during infection progression. These data provide a temporal framework to examine how sustained host defense responses are transcriptionally coordinated with progressive changes in core physiological and metabolic processes during MoT infection.

## 2 Materials and methods

### 2.1 Plant growth and fungal growth conditions

The MoT isolate BTJP, originally collected in Bangladesh, was maintained in the laboratory of N.J.T. at The Sainsbury Laboratory following previously described protocols (Talbot et al., 1993). Conidia were harvested from 10-day-old cultures grown on complete medium agar plates at 24 °C. For infection assays, conidia were used to inoculate the spring wheat cultivar Cadenza. Infected and control wheat plants were incubated in a controlled growth chamber at 24 °C with a 12-hour photoperiod. Illumination was provided by a metal halide lamp (Osram Powerstar HQI-Bt 400 W/D) at an intensity of 500 μmol m^−2^ s^−1^, and relative humidity was maintained at 90% to facilitate fungal infection.

### 2.2 Wheat infection assay and sample collection for RNA sequencing

Wheat seeds were pregerminated and sown in cell trays, then cultivated under controlled environmental conditions with a long-day photoperiod (16 h light/8 h dark) and a day/night temperature cycle of 22 °C/18 °C. After seedling establishment under standard growth conditions, plants were transferred to a controlled infection chamber immediately following inoculation to ensure uniform and reproducible disease development. For pathogen inoculation, a conidial suspension (1 × 10^6^ spores mL^-1^ in 0.25% (w/v) gelatin) was prepared. Twenty microliters of the suspension were applied to the leaves of 8-day-old wheat seedlings by spot inoculation. Mock-inoculated plants were treated with the same volume of 0.25% gelatin solution without spores to control for any potential inoculation effect. Inoculation droplets were allowed to wick naturally during the first 24 hpi.

### 2.3 RNA extraction and sequencing

Leaf spot samples were collected at 0, 24, 36, and 48 hpi for RNA extraction. Samples were immediately frozen in liquid nitrogen and ground into a fine powder using a mortar and pestle. Total RNA was extracted using the TRIzol Reagent (Invitrogen, USA) according to the manufacturer’s instructions. RNA concentration and purity were measured using a Nanodrop spectrophotometer (Thermo Fisher Scientific, USA). RNA integrity was assessed using an Agilent 2100 Bioanalyzer (Agilent Technologies, USA), with an average RNA Integrity Number (RIN) of 6.5. This level is consistent with published evidence demonstrating that RNA-sequencing (RNA-seq) can generate reliable differential expression results even from partially degraded RNA, provided that RNA quality is not confounded with experimental groups (Gallego Romero et al., 2014). Raw sequencing reads were generated as paired-end 150 bp (PE150) on the Illumina NovaSeq X platform. Three independent biological replicates were included for each time point and treatment. Each replicate consisted of leaves collected from different wheat plants, which were randomly assigned and inoculated independently, and samples were processed individually without pooling.

### 2.4 Read processing and differential gene expression analysis

Raw sequencing data were deposited in the National Center for Biotechnology Information (NCBI) BioProject database under accession number PRJNA1392009. Quality control and adapter trimming were performed using fastp (v0.20.1), with automatic adapter detection enabled for both R1 and R2 reads. Sliding-window trimming was applied to both 5’ and 3’ ends, using a 4-bp window; bases with a mean Phred quality score below 20 within the window were trimmed. Reads shorter than 75 bp after trimming or containing more than 5% ambiguous bases (N) per read were discarded. The quality of clean reads was evaluated using FastQC (v0.11.9) and RSeQC (v4.0.0). Detailed statistics for raw and clean reads, including Q30 percentages, valid bases, and GC content, are provided in **Table 1**.

**Table 1:** Summary of sequencing depth and quality metrics for all RNA-seq libraries used in this study.

Sample	RawReads (M)	RawBases (G)	CleanReads (M)	CleanBases (G)	ValidBases (%)	Q30 (%)	GC (%)
INF_0h-1	43.39	6.43	41.91	6.21	96.59	95.45	55.00
INF_0h-2	41.31	6.16	40.65	6.06	98.41	97.77	56.13
INF_0h-3	44.30	6.60	43.32	6.45	97.79	96.74	56.14
INF_24h-1	41.32	6.15	40.69	6.06	98.49	98.26	54.24
INF_24h-2	41.18	6.13	40.60	6.05	98.59	98.09	54.12
INF_24h-3	47.37	7.06	46.64	6.95	98.46	97.44	53.32
INF_36h-1	49.23	7.33	48.41	7.21	98.33	97.25	53.22
INF_36h-2	42.18	6.29	41.45	6.18	98.26	96.58	53.50
INF_36h-3	44.88	6.69	44.20	6.59	98.49	97.47	52.31
INF_48h-1	66.46	9.92	65.78	9.82	98.97	98.21	54.45
INF_48h-2	46.08	6.87	45.36	6.76	98.43	97.00	54.14
INF_48h-3	40.77	6.08	40.22	6.00	98.67	97.85	54.20
CK_0h-1	48.43	7.22	47.71	7.12	98.52	97.05	56.29
CK_0h-2	39.66	5.91	38.71	5.77	97.61	96.02	55.49
CK_0h-3	43.84	6.52	42.37	6.30	96.66	94.67	55.91
CK_24h-1	47.37	7.07	46.62	6.96	98.42	96.78	53.37
CK_24h-2	49.46	7.34	47.86	7.10	96.76	96.32	52.31
CK_24h-3	47.09	7.02	46.47	6.93	98.68	97.89	52.99
CK_36h-1	41.01	6.11	40.47	6.03	98.67	98.37	52.79
CK_36h-2	42.32	6.30	41.53	6.19	98.14	97.38	52.90
CK_36h-3	41.77	6.21	40.61	6.04	97.23	95.93	52.88
CK_48h-1	50.87	7.57	49.77	7.41	97.84	96.73	52.55
CK_48h-2	41.11	6.13	40.19	5.99	97.77	96.07	52.92
CK_48h-3	57.99	8.64	56.94	8.48	98.19	96.94	52.77

RawReads and RawBases indicate the total number of sequencing reads and bases before quality filtering. CleanReads and CleanBases represent reads retained after adaptor removal and quality trimming. ValidBases (%) refers to the proportion of bases passing quality control. Q30 (%) indicates the percentage of bases with Phred quality scores ≥30. GC (%) represents the guanine–cytosine content of the clean reads. INF and CK denote MoT-inoculated and mock-inoculated samples, respectively.

Clean reads were aligned to the *Triticum aestivum* reference genome (IWGSC RefSeq v1.0) and *Magnaporthe oryzae* pathotype *Oryzae* strain 70–15 genome (GCA_000002495) using HISAT2 (v2.1.0) with default parameters. The *Magnaporthe oryza*e pathotype Oryzae (MoO) genome was used because the MoT B71 genome lacks comprehensive annotation. Despite this limitation, the B71 genome exhibits high macrosynteny with the MoO genome, consistent with their classification as a single species (Gladieux et al., 2018; Peng et al., 2019). Previous transcriptomic studies of the MoT strain B71 have likewise used the annotated MoO MG8 genome assembly (GCA_000002495) for gene expression analysis (Peng et al., 2019). Consistent with prior transcriptomic studies, reads were aligned to the MG8 genome and obtained fungal mapping rates of 4.62%–9.04% (excluding the 0 hpi controls). These fungal reads provided the basis for downstream effector prediction and functional annotation.

Sequencing reads generated from infected wheat leaves were mapped independently to the wheat and fungal reference genomes using HISAT2, which assigns mapping quality (MAPQ) scores to each alignment. Only uniquely mapped reads were retained to exclude ambiguous placements and ensure conservative assignment of host and pathogen transcripts.

BAM files were sorted and indexed using SAMtools (v1.9) for downstream differential expression analysis. Gene-level counts were obtained using HTSeq-count (v0.11.2) in union mode. Differential expression analysis was performed using DESeq2 (v1.22.2), comparing transcriptomic changes across successive infection stages (0, 24, 36, and 48 hpi) and between inoculated and mock-treated samples. Wheat genes with an adjusted *p-*value < 0.05 and an absolute log_2_ fold change > 1 were considered significantly differentially expressed for downstream analyses, as well as MoT genes. Principal component analysis (PCA) was conducted on all expressed wheat genes using the prcomp function in R to assess global transcriptome variance and to evaluate clustering patterns among infection stages and biological replicates. Differentially expressed genes (DEGs) were visualized using heatmaps generated with the ComplexHeatmap and ggplot2 packages in R.

### 2.5 Functional enrichment analysis

Gene Ontology (GO) enrichment analysis was performed to investigate the functional roles of DEGs in the Biological Process (BP), Molecular Function (MF), and Cellular Component (CC). GO terms with an adjusted *p-*value < 0.05 were considered significantly enriched. Kyoto Encyclopedia of Genes and Genomes (KEGG) pathway enrichment analysis was performed using the KEGG database. DEGs were mapped to known signaling and metabolic pathways, and enrichment significance was evaluated using a hypergeometric test with multiple testing correction, and terms with adjusted p-values < 0.05 were considered significant. Enriched GO terms and KEGG pathways were interpreted in the context of host defense responses, metabolic reprogramming, and wheat blast susceptibility.

### 2.6 In silico prediction and annotation of MoT effector candidates

Open reading frames (ORFs) of each sequence (≥100 amino acids) were first extracted using TransDecoder v5.7.0. Functional annotation was supported by domain-based and homology-based evidence. Predicted peptides were scanned against the Pfam-A database (HMMER v3.3.2, E-value ≤ 1e−5) to identify conserved protein domains, and sequence similarity searches were conducted against the NCBI non-redundant (NR) database using DIAMOND v2.1.9 (blastp mode, E-value ≤ 1e−5). The Pfam and DIAMOND results were integrated into TransDecoder to retain the most biologically relevant ORFs.

Fungal effectors typically share several key features, including signal peptides, low molecular weight, and high cysteine content (Selin et al., 2016). Proteins with predicted signal peptides (SignalP 5.0) and no transmembrane domains (DeepTMHMM 1.0) were filtered for extracellular space or plasma membrane localization using DeepLoc 2.1 (Almagro Armenteros et al., 2019; Hallgren et al., 2022; Ødum et al., 2024). Glycosylphosphatidylinositol (GPI) anchored proteins were identified using NetGPI 1.1, and GPI-anchored proteins were excluded (Gíslason et al., 2021). The remaining sequences were analyzed using EffectorP v3.0 to predict effector candidates (Sperschneider and Dodds, 2021), and CAZyme-containing proteins were excluded based on dbCAN3 predictions (Zheng et al., 2023). The final set of effector candidates comprised proteins containing a signal peptide, no transmembrane domains or GPI anchor, localized to the extracellular space or plasma membrane, and devoid of containing carbohydrate-active enzymes (CAZymes). The number of cysteine residues of each candidate protein sequence was counted, and the cysteine ratio was calculated as a percentage of the mature sequence length. Expression profiles of predicted effector candidates were subsequently integrated with transcriptome data to assess their temporal expression patterns during infection.

### 2.7 Co-expression network analysis of wheat and MoT genes

Functional annotation of effector candidates, including protein domains and GO enrichment analysis, was performed using DAVID Bioinformatics Resources (Huang et al., 2009; Sherman et al., 2022). Expression matrices for MoT and wheat were generated separately from RNA-seq reads mapped to respective genomes, and then combined for cross-species correlation analysis. Expression data were used to generate a Z-score heatmap of normalized transcript levels, with annotations corresponding to the GO term GO:0008061 (chitin binding), which is associated with fungal recognition and host defense responses. Normalization was performed separately for each species using Z-scores across time points to allow meaningful cross-species correlation analysis. The co-expression relationships between MoT and wheat genes were analyzed using Pearson correlation across all four sampled time points (0, 24, 36, and 48 hpi). A stringent correlation threshold (|r| ≥ 0.8) was applied to define high-confidence co-expression relationships. This conservative cutoff was chosen to reduce spurious associations inherent to short time-series transcriptomic designs. Correlation coefficients were used to identify robust co-expression patterns suitable for bipartite network visualization. Therefore, multiple testing correction was not applied because the goal was pattern detection rather than statistical inference. A bipartite graph was constructed, with nodes representing genes from both species and edges indicating significant correlations. The network was visualized using bipartite network layouts, with edge weights reflecting the strength of the correlations. All visualizations were generated using R packages, including igraph, ggraph, ComplexHeatmap, and ggplot2.

## 3 Results

### 3.1 Temporal dynamics of wheat blast symptom development inform the sampling window

**Figure 1:**
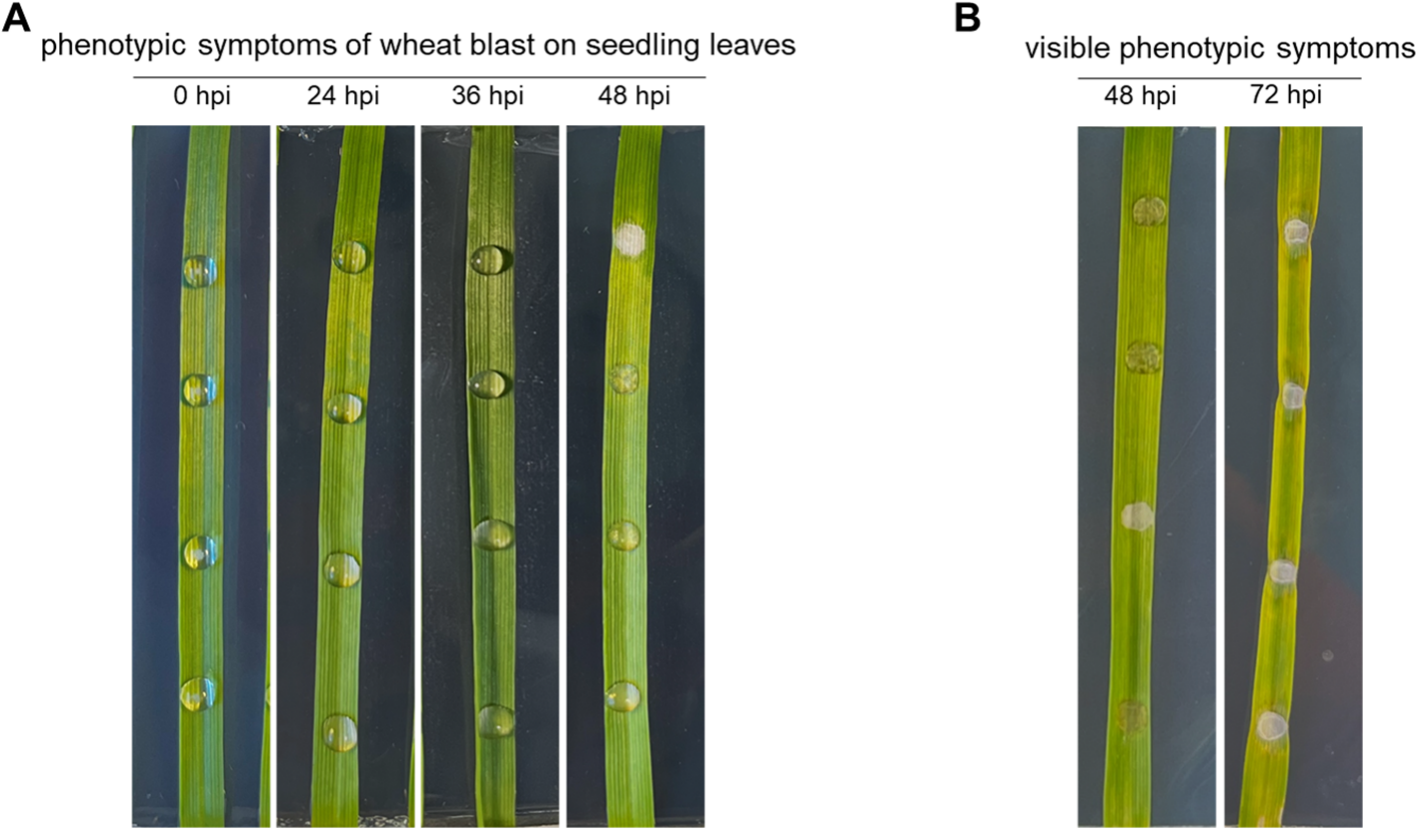
Disease progression of wheat blast on seedling leaves of the winter wheat cultivar Cadenza. **(A)** Seedling leaves at 0, 24, 36, and 48 hpi following leaf-drop inoculation with MoT. These time points correspond to the transcriptome sampling stages. **(B)** Representative disease symptoms at 48 and 72 hpi. The 72 hpi time point is shown to illustrate lesion development but was excluded from transcriptome analysis due to extensive tissue necrosis and RNA degradation.

To characterize transcriptional responses associated with wheat blast disease, we selected the susceptible wheat cultivar ‘Cadenza’ for transcriptomic analysis at 0, 24, 36, and 48 hpi. We specifically included the 36 and 48 hpi time points to capture transcriptional changes associated with the progression from the earliest sampled stage to pre-necrotic symptom development, as shown in **Figure 1A**. Sampling was designed to capture early host transcriptional responses associated with infection while minimizing confounding effects from extensive tissue damage. The Cadenza cultivar is widely used in wheat research, particularly as the genotype for the TILLING Exon Capture Population, a valuable genetic resource for functional studies (Krasileva et al., 2017; Fernández-Gómez et al., 2020). Although its susceptibility to wheat blast has not been extensively characterized, our observations showed that ‘Cadenza’ developed typical wheat blast lesions on leaves following MoT infection.

Following leaf-drop inoculation, we monitored symptom development on seedling leaves over time. This aligns with a previous RNA-seq study on MoO, which reported that primary infection hyphae penetrate leaf epidermal cells at approximately 24 hpi (Kawahara et al., 2012). Accordingly, 24 hpi was chosen as the earliest sampling point to capture host transcriptional responses associated with early infection, corresponding to the reported penetration stage of MoO and used here as a temporal reference for early infection.

By 48 hpi, the inoculated conidial suspension had completely dried, and infected leaf regions exhibited water-soaking and mild chlorosis (**Figures 1A, B**). At 72 hpi, characteristic elliptical lesions with white to tan centers and necrotic brown margins became evident, confirming disease progression (**Figure 1B**). However, RNA extracted from 72 hpi samples did not meet sequencing quality requirements, likely due to tissue necrosis and RNA degradation. Consequently, we identified the 0–48 hpi window as the optimal period for initial to pre-necrotic transcriptional responses, prior to visible necrosis.

### 3.2 Transcriptome sequencing and quality assessment of MoT-infected wheat seedling leaves

Transcriptome sequencing was performed on leaf samples collected at 0, 24, 36, and 48 hpi, generating 24 mRNA libraries across these four time points. Illumina high-throughput sequencing generated a total of 160.66 Gb of clean data (**Table 1**). After quality filtering, each sample yielded 5.77–9.82 Gb of clean reads, with 94.67%–98.37% of bases above Q30 and an average GC content of 53.91%, indicating consistent high sequencing quality across all samples.

Clean reads were aligned to the *Triticum aestivum* reference genome (IWGSC RefSeq v1.0), achieving mapping rates of 85.88%–97.15%. Reads were also aligned to the MoO strain 70–15 genome for pathogen analysis. This approach allows assessment of global fungal transcriptional trends, but some MoT-specific effectors may not be captured due to differences between MoO and MoT repertoires, reflecting a conservative effector discovery framework. Wheat and fungal reads were mapped independently, and only uniquely mapped reads were retained to exclude ambiguous alignments and ensure conservative host-pathogen expression profiling.

### 3.3 Overview of differentially expressed genes during MoT infection in wheat leaves

**Figure 2:**
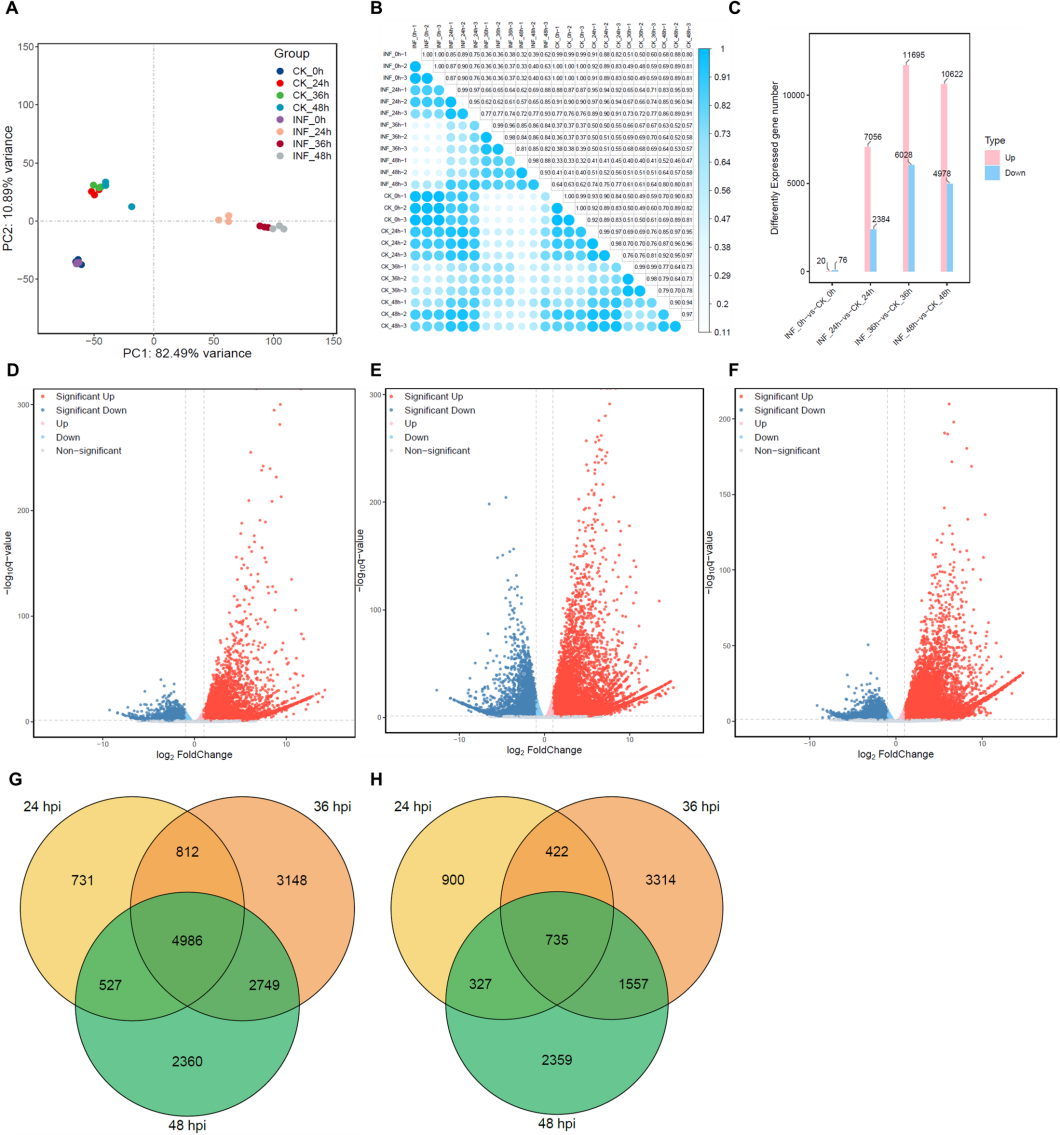
Transcriptional responses and DEG architecture of wheat leaves during MoT infection. **(A)** Principal component analysis separates mock-inoculated and MoT-infected samples across all time points, showing tight clustering of biological replicates. **(B)** Heatmap of pairwise Pearson correlations based on normalized TPM values confirms high reproducibility within each condition and time point. **(C)** Numbers of significantly upregulated (pink) and downregulated (blue) genes at each infection stage between mock-inoculated (CK) and infected (INF) samples. Comparisons were also made between successive stages (log_2_ fold change > 1, adjusted p < 0.05). **(D–F)** Volcano plots showing DEGs between mock-inoculated and MoT-infected samples at 24 hpi **(D)**, 36 hpi **(E)**, and 48 hpi **(F)**. Upregulated genes are shown in red, downregulated genes in blue. **(G, H)** Venn diagrams illustrating the overlap of upregulated **(G)** and downregulated **(H)** genes infection stages. Circles are color-coded by time point: yellow (24 hpi), orange (36 hpi), and green (48 hpi), highlighting both shared and stage-specific transcriptional responses to MoT infection.

Transcript abundance was quantified as transcripts per million (TPM) for expression visualization and cross-sample comparison. Differential expression analysis was performed on raw gene counts using DESeq2 with its default normalization framework. Principal component and correlation analyses based on normalized expression values from all expressed genes revealed clear separation among infection stages, with PC1 explaining 82.49% of the variance and PC2 explaining 10.89%. Biological replicates clustered tightly within each group, indicating strong transcriptomic structure and high data reproducibility (**Figures 2A, B**).

Differential expression was defined using a significance threshold of adjusted *p*-value (padj) < 0.05 combined with an absolute log_2_ fold change (|log_2_FC|) > 1. Under this threshold, 7,056 genes were upregulated and 2,384 were downregulated at 24 hpi relative to mock-inoculated controls (CK_24 h vs. INF_24 h) (**Figures 2C, D**), consistent with early defense activation and redox buffering. The number of differentially expressed genes (DEGs) peaked at 36 hpi, with 11,695 upregulated and 6,028 downregulated under the same significance threshold (CK_36 h vs. INF_36 h) (**Figures 2C, E**). At 48 hpi, 10,622 upregulated and 4,978 downregulated genes were identified using the same criteria (CK_48 h vs. INF_48 h) (**Figures 2C, F**). To identify genes with consistent versus stage-specific transcriptional responses to MoT infection, we generated Venn diagrams of DEGs at 24, 36, and 48 hpi (**Figures 2G, H**). Separate diagrams for upregulated and downregulated genes revealed that a subset of genes was consistently upregulated or downregulated across all time points, while many others showed stage-specific expression. Notably, a core set of genes remained consistently upregulated across all infection stages, suggesting sustained immune activation. Additionally, 20 upregulated and 76 downregulated genes were identified at 0 hpi (**Supplementary Figure 2**). These differences likely reflect baseline transcriptional variation associated with sample handling or inoculation procedures, rather than pathogen-driven responses. Therefore, no functional enrichment was performed for this comparison. Across all three infection time points, upregulated genes consistently outnumbered repressed genes, indicating that MoT infection triggered a broadly upregulated transcriptional response rather than uniform transcriptional downregulation. This pattern is consistent with rapid mobilization of defense and detoxification-related transcriptional programs during pathogen infection (Kabbage et al., 2015). A comprehensive list of DEGs is provided in **Supplementary Table 2**.

**Figure 3:**
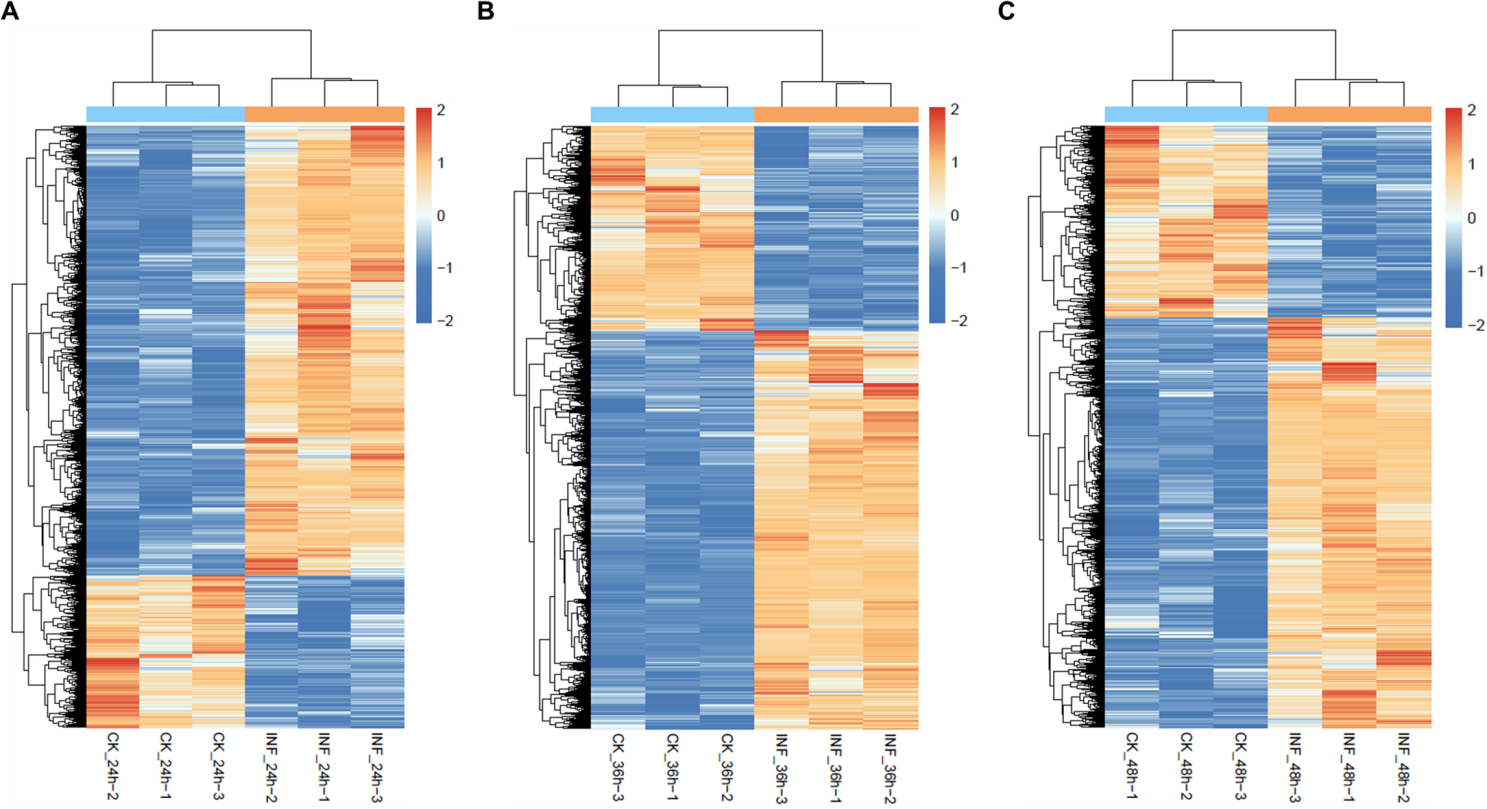
Time-dependent transcriptional reprogramming of wheat genes during MoT infection. **(A–C)** Hierarchical clustering heatmaps of differentially expressed genes at 24 hpi **(A)**, 36 hpi **(B)**, and 48 hpi **(C)**. Mock- and MoT-inoculated samples display distinct expression clusters at each infection stage. Sample labels indicate treatment, time point, and biological replicate. Gene expression values were Z-score normalized across samples, with the color scale ranging from −2 to 2.

Hierarchical clustering of DEGs across the three time points (**Figures 3A–C**) revealed clear distinctions between MoT-inoculated and mock-treated samples, with consistent patterns among biological replicates. Overall, MoT infection triggered broad, time-dependent transcriptional reprogramming, prompting further functional analysis through GO and KEGG enrichment.

### 3.4 Temporal functional enrichment reveals distinct phases of host responses to MoT infection

**Figure 4:**
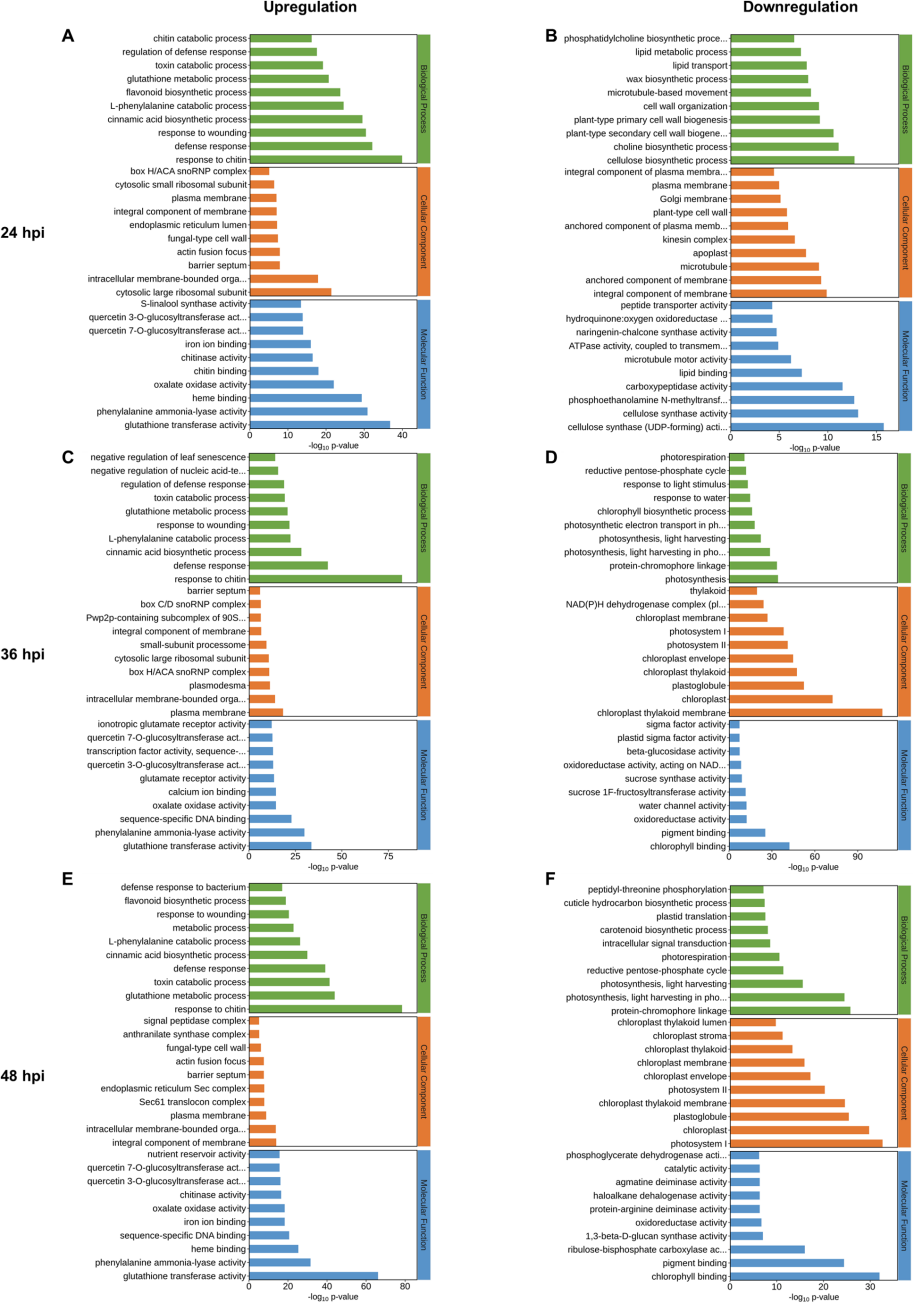
Temporal GO enrichment of wheat differentially expressed genes during MoT infection. GO enrichment analysis was performed for DEGs at 24, 36, and 48 hpi. The top 10 terms from each GO category are shown for upregulated and downregulated genes, including Biological Process (BP, green), Cellular Component (CC, orange), and Molecular Function (MF, blue). Terms are ranked by −log10(p-value) on the x-axis, with GO term names on the y-axis. Each panel displays 30 terms in total. **(A, B)** GO enrichment patterns for upregulated **(A)** and downregulated **(B)** genes at 24 hpi. **(C, D)** GO enrichment patterns for upregulated **(C)** and downregulated **(D)** genes at 36 hpi. **(E, F)** GO enrichment patterns for upregulated **(E)** and downregulated **(F)** genes at 48 hpi.

**Figure 5:**
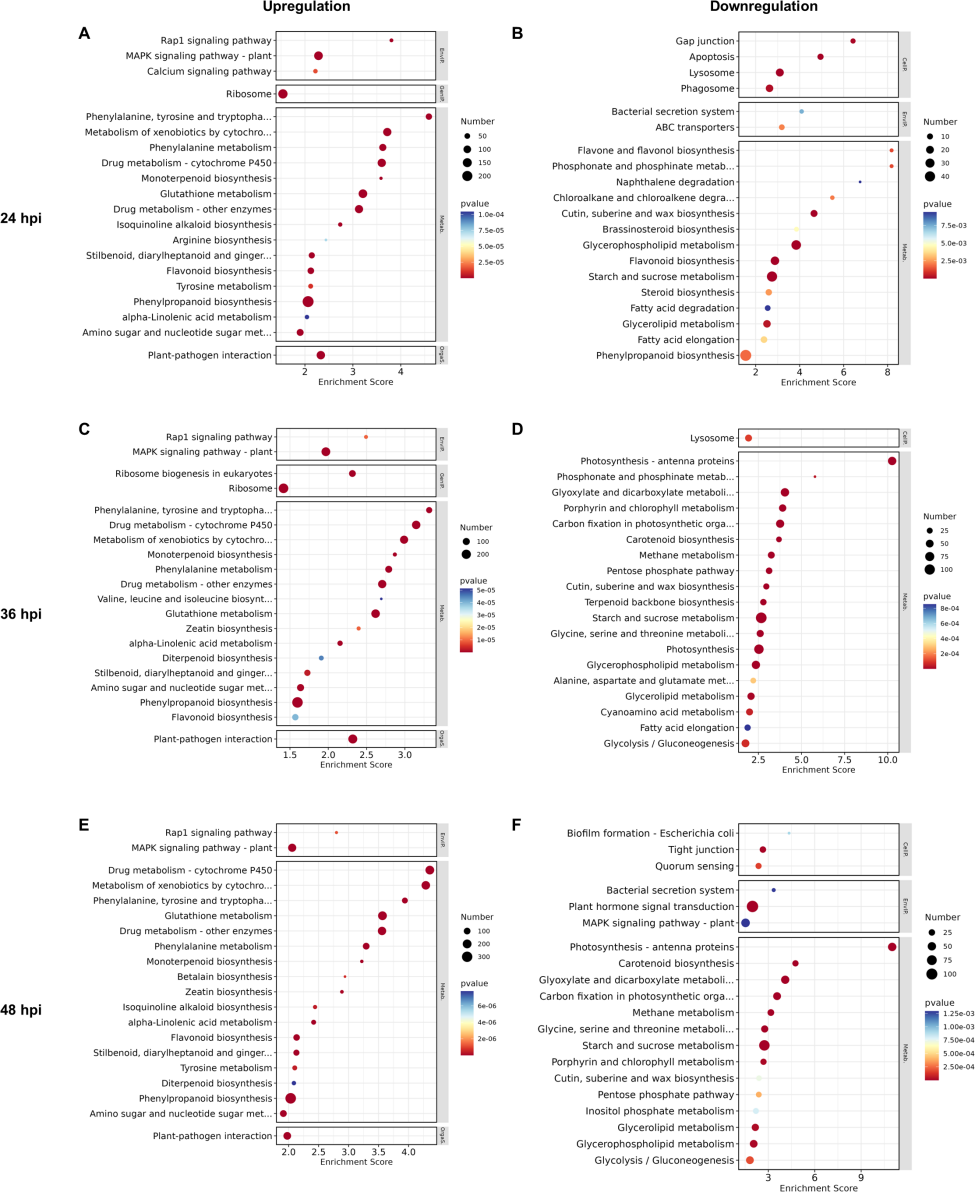
KEGG pathway enrichment highlights stage-specific transcriptional reprogramming during MoT infection. Top KEGG pathways enriched among DEGs at 24, 36, and 48 hpi. Bubble size indicates the number of genes mapped to each pathway, and color intensity represents −log10(p-value). Pathways are categorized into Cellular Processes (CellP), Environmental Information Processing (EnvIP), Organismal Systems (OrgaS) and Metabolism (Metab), as indicated by colored boxes on the right side of each panel. **(A, B)** Upregulated **(A)** and downregulated **(B)** genes at 24 hpi. **(C, D)** Upregulated **(C)** and downregulated **(D)** genes at 36 hpi. **(E, F)** Upregulated **(E)** and downregulated **(F)** genes at 48 hpi.

GO enrichment analysis was performed on DEGs at 24, 36, and 48 hpi to characterize functional transitions during wheat blast infection, with enriched terms classified into Biological Process (BP), Molecular Function (MF), and Cellular Component (CC) categories (**Supplementary Table 3**). The top ten significantly enriched GO terms in each category for both up- and downregulated genes are shown in **Figure 4**. To place these GO terms into broader biological context, KEGG pathways enrichment was performed in parallel, and the top twenty enriched pathways for both upregulated and downregulated genes at each time point are shown in **Figure 5**, with complete results provided in **Supplementary Table 4**.

At 24 hpi, upregulated genes were predominantly enriched in GO terms associated with immune responses and xenobiotic processing, including response to chitin, defense responses, response to wounding, and flavonoid biosynthesis, accompanied by increased chitinase activity and glutathione S-transferase (GST) activity (**Figure 4A**). KEGG analysis further supported these patterns, showing enrichment of glutathione metabolism, cytochrome P450-mediated detoxification, mitogen-activated protein kinases (MAPK) signaling, and phenylpropanoid and flavonoid biosynthesis (**Figure 5A**). In contrast, downregulated genes were enriched for cell wall- and membrane-associated processes, including cellulose biosynthetic process, cellulose synthase activity, choline biosynthetic process, and microtubule-associated terms (**Figure 4B**), with KEGG revealing enrichment of glycerophospholipid metabolism and cutin, suberin, and wax biosynthesis among downregulated genes (**Figure 5B**).

At 36 hpi, upregulated DEGs remained enriched in immune- and detoxification-related pathways, accompanied by coordinated transcriptional downregulation of chloroplast- and photosynthesis-associated genes, indicating a defense-associated transcriptional state accompanied by coordinated metabolic downscaling. Upregulated DEGs were enriched in canonical defense- and stress-related GO terms, including response to chitin, defense responses, and glutathione metabolic process (**Figure 4C**). Consistently, KEGG analysis identified enrichment of cytochrome P450-mediated xenobiotic metabolism, glutathione metabolism, MAPK signaling pathways, phenylpropanoid biosynthesis, and alpha-linolenic acid metabolism (**Figure 5C**). In contrast, downregulated DEGs were enriched for chloroplast- and photosynthesis-related GO terms, photosynthesis, photosystem I light harvesting, photosystem I and II components, chloroplast thylakoid membranes, pigment binding, and the reductive pentose-phosphate cycle (**Figure 4D**). Consistently, KEGG analysis revealed suppression of photosynthesis-antenna proteins, carbon fixation, porphyrin and chlorophyll metabolism, starch and sucrose metabolism, and glyoxylate pathways (**Figure 5D**). These findings are consistent with the emergence of coordinated transcriptional repression of photosynthesis- and chloroplast-associated genes at 36 hpi, concurrent with sustained transcriptional activation of immune- and detoxification-related pathways.

At 48 hpi, when water-soaking and chlorosis became visible (**Figure 1**), enrichment patterns intensified relative to 36 hpi, reflecting more extensive transcriptional remodeling during active symptom development. Upregulated DEGs were predominantly enriched in immunity- and detoxification-related GO terms, including response to chitin, glutathione metabolic process, toxin catabolic process, and defense responses, together with molecular functions such as phenylalanine ammonia-lyase activity, glutathione transferase activity, and chitinase activity (**Figure 4E**). Consistently, KEGG analysis revealed strong enrichment of cytochrome P450–mediated xenobiotic metabolism, glutathione metabolism, phenylpropanoid and flavonoid biosynthesis, as well as multiple secondary metabolic branches including stilbenoid, monoterpenoid, diterpenoid, and isoquinoline alkaloid biosynthesis, together with MAPK signaling and alpha-linolenic acid metabolism (**Figure 5E**). In contrast, downregulated DEGs were strongly enriched for chloroplast- and photosynthesis-related GO terms, including light-harvesting in photosystem I, photosystem I and II components, reductive pentose phosphate cycle, photorespiration, chloroplast thylakoid membrane, chloroplast stroma, and chlorophyll and pigment binding (**Figure 4F**). KEGG analysis further revealed suppression of photosynthesis-antenna proteins, carbon fixation in photosynthetic organisms, porphyrin and chlorophyll metabolism, carotenoid biosynthesis, starch and sucrose metabolism, glyoxylate and dicarboxylate metabolism, and the pentose phosphate pathways (**Figure 5F**). At 48 hpi, wheat transcriptomes exhibited sustained activation of defense and detoxification pathways together with broad transcriptional repression of photosynthesis, chloroplast maintenance, and primary carbon metabolism coinciding with lesion initiation.

Collectively, the temporal functional enrichment profiles delineate distinct phases of host responses: an early activation of defense and detoxification pathways accompanied by cell wall and membrane remodeling at 24 hpi, followed by progressive repression of chloroplast- and photosynthesis-associated processes together with sustained activation of immune- and detoxification-related pathways at 36–48 hpi. These results outline a distinct temporal trajectory of host transcriptional responses, characterized by progressive activation of immune- and detoxification-related pathways and coordinated repression of photosynthesis-associated functions as disease symptoms develop.

### 3.5 Transcriptome-based prediction of secreted effector candidates in MoT

To identify secreted effector candidates expressed by MoT during infection, we performed a transcriptome-based prediction analysis based on the annotated MoO reference genome, enabling the detection of MoT transcripts corresponding to genes with annotated orthologs in MoO. From the MoT transcriptome, we predicted a total of 14,477 proteins, of which 1,583 proteins (10.93%) contained signal peptides, suggesting they may function as secreted proteins. From this subset, we selected 1,417 mature proteins that lacked transmembrane helical segments for further analysis. These proteins were then filtered based on their predicted organelle localization. Next, 1,144 proteins were examined, and those with glycosylphosphatidylinositol (GPI) anchors were excluded from further analysis. After removing GPI-anchored proteins, 1,001 non-GPI-anchored proteins remained. Among these, 459 proteins were retained based on EffectorP 3.0 predictions. From the 459 proteins, we excluded those containing carbohydrate-active enzymes (CAZymes), resulting in a final set of 390 MoT effector candidates, including three genes with isoforms (MGG_05883, MGG_07606, and MGG_08296), yielding a total of 393 protein variants (**Table 2** and **Supplementary Table 1**). Of these, 251 effector candidates were classified as cysteine-rich proteins (with ≥4 cysteine residues) (Klosterman et al., 2011).

**Table 2:** Summary of predicted MoT effector candidates and filtering steps.

Filtering step	Criteria	Number of proteins
Total predicted proteins	—	14,477
Secreted proteins	Signal peptide (Sec/SPI)	1,583
Without transmembrane domains	No predicted TM helices	1,417
Subcellular localization	Extracellular space or plasma membrane	1,141
GPI-anchor filtering	Non-GPI-anchored proteins	1,001
Effector prediction	EffectorP v3.0	459
CAZyme filtering	Non-CAZyme proteins	393
**Final effector candidates**	Summary of retained candidates	**393** (from 390 genes, including isoforms)

Proteins were sequentially filtered based on secretion signal peptides, transmembrane domains, subcellular localization, GPI-anchor prediction, effector prediction, and CAZyme exclusion. The final effector set comprises 393 protein variants derived from 390 genes, including isoforms.

Bold text indicates the final effector candidate set (393 proteins derived from 390 genes, including isoforms) after sequential filtering.

Notably, our list of effector candidates includes well-characterized *M. oryzae* effectors, including Pwl1, Pwl2 (Kang et al., 1995), MoCDIPs (Guo et al., 2019), MoHrip1, MoHrip2 (Nie et al., 2019; Yan et al., 2025), SPD5 (Sharpee et al., 2017), SPD11,Nup1, and *Avr*-Pita (Chuma et al., 2011), along with other previously identified effectors, which further validate the reliability of our prediction pipeline.

**Figure 6:**
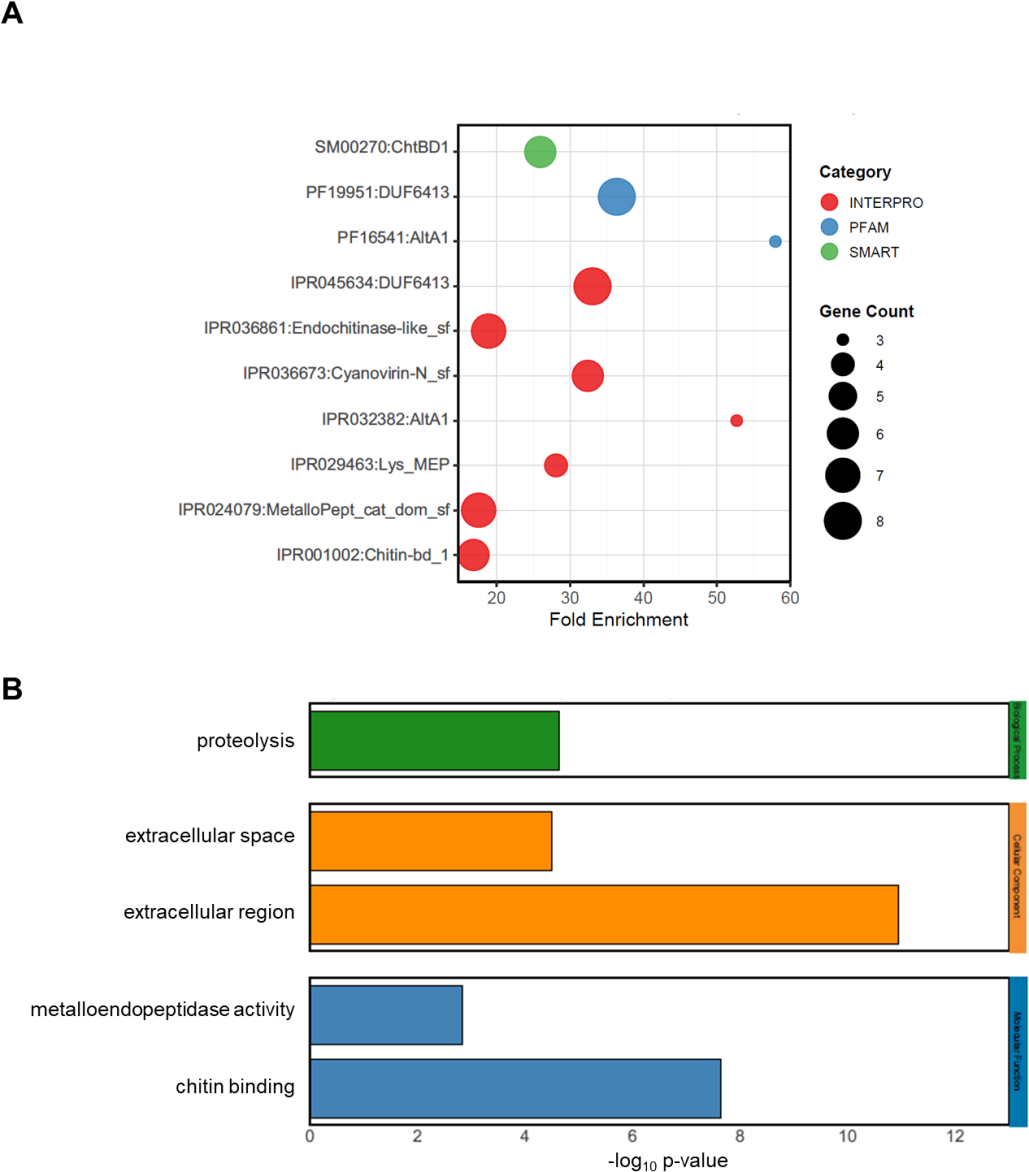
Functional annotation and domain composition of predicted MoT effector candidates. **(A)** Domain architecture of predicted effectors based on InterPro (red), Pfam (blue), and SMART (green). Bubble size indicates the number of genes associated with each domain. All displayed domains have FDR < 0.05. Domains detected in multiple databases are shown separately for clarity. **(B)** GO enrichment of predicted effector candidates. Significantly enriched terms from Biological Process (BP, green), Cellular Component (CC, orange), and Molecular Function (MF, blue) categories are shown, highlighting the predominant functional categories of effector candidates.

To explore the potential functions of effector candidates, we annotated protein domains and GO terms using the DAVID database (**Supplementary Table 1** and **Supplementary Table 3**). Domain annotation revealed that six effector candidates contained chitin-binding domains, as supported by both SMART and InterPro databases (**Figure 6A**). Among these, MGG_12049 was annotated as belonging to the endochitinase-like superfamily. In addition, another effector candidate, MGG_07571, was enriched in chitin-binding GO terms (**Figure 6B**). Additionally, the DUF6413 domain (protein of unknown function) was annotated in eight effector candidates according to both PFAM and InterPro databases. The functions and mechanisms of these DUF6413-containing proteins remain poorly understood and warrant further investigation. Three candidates contained the *Alternaria alternata* allergen 1 (Alt a 1; AA1) domain from PFAM and InterPro databases, including MoHrip1, which belongs to the AA1 family (Zhang et al., 2017). Six effector candidates contained the cyanovirin-N (CV-N) domain, derived from the InterPro database. Another seven effector candidates belonged to the metallopeptidase catalytic domain superfamily, which likely represents the catalytic domain of various metallopeptidases (Cerdà-Costa and Gomis-Rüth, 2014). Four of these candidates, including *Avr*-Pita1, contained a lysine-specific metallo-endopeptidase domain, which was also enriched with metallo-endopeptidase activity GO terms (**Figures 6A, B**). The extracellular region, extracellular space, and proteolysis GO terms were also significantly enriched, serving as characteristics of many effectors in **Figure 6B** (Langin et al., 2020). These functional annotations indicate that MoT effector candidates encompass diverse predicted biochemical activities, including manipulation of host chitin-binding proteins and catalytic mechanisms involving metal ions.

### 3.6 Co-expression analysis between MoT and wheat genes binding to chitin

**Figure 7:**
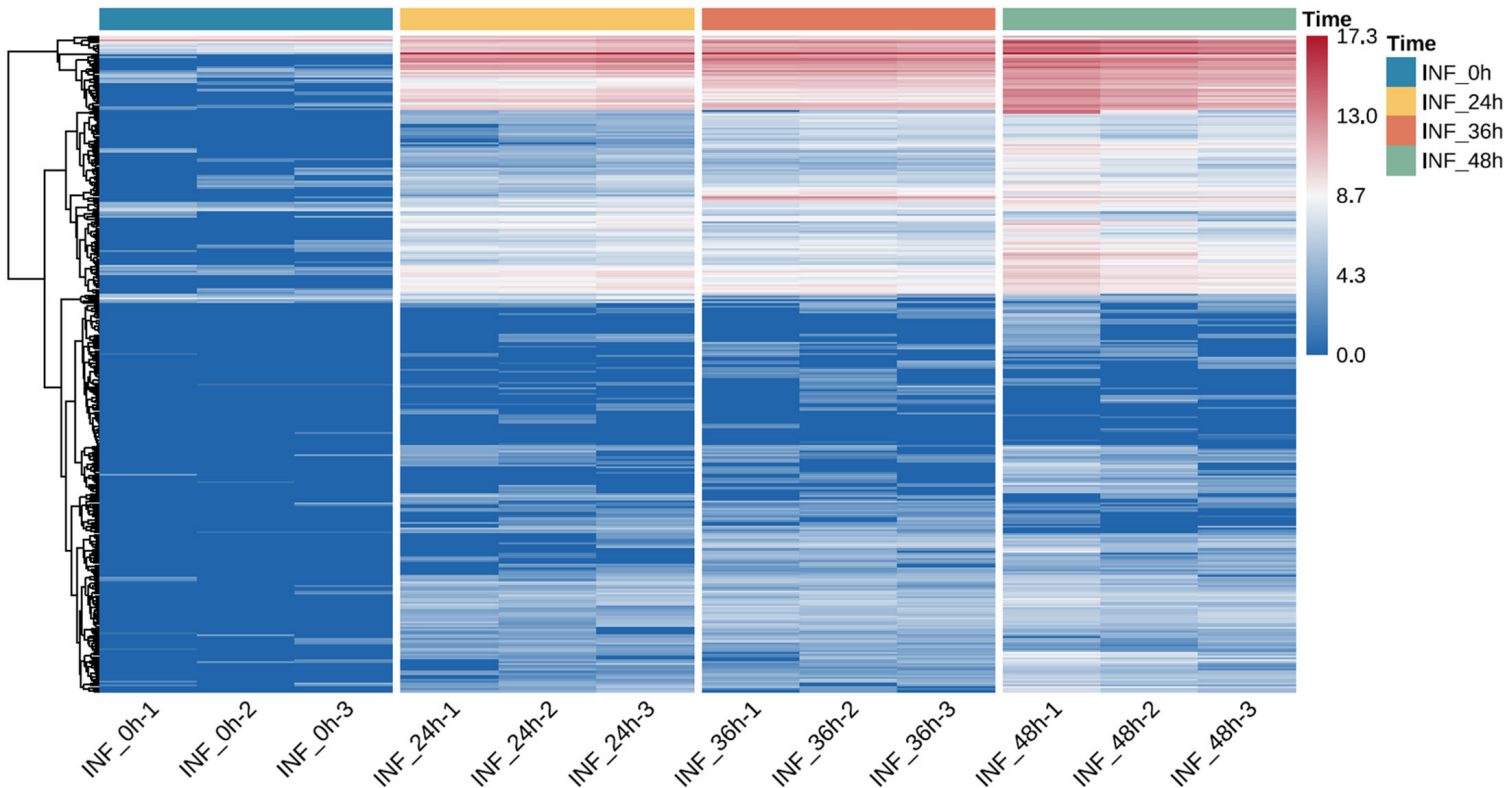
Temporal expression patterns of predicted MoT effector candidates during wheat infection. Heatmap showing expression dynamics of predicted MoT effector candidates across infection stages (0, 24, 36, 48 hpi) in infected leaves. Only inoculated samples are included. Gene expression values are log_2_ (TPM + 1). Rows represent effector candidates, and columns represent samples ordered by infection time. Rows and columns were hierarchically clustered to highlight temporal expression dynamics. Column annotation indicates infection time points, with distinct colors representing each stage.

Among 390 effector candidates, 47 showed no expression (TPM = 0) across all four time points (0, 24, 36, and 48 hpi; **Figure 7**). Among the remaining candidates, 80 showed consistent upregulation across three time points (24, 36, and 48 hpi). Of these, 13 suggested sustained and significant upregulation. The number of significantly upregulated effector candidates increased progressively during infection: 90 at 24 hpi, 123 at 36 hpi, and 131 at 48 hpi. This temporal increase in effector expression is consistent with dynamic transcriptional activation as infection advances from initial colonization toward lesion formation.

**Figure 8:**
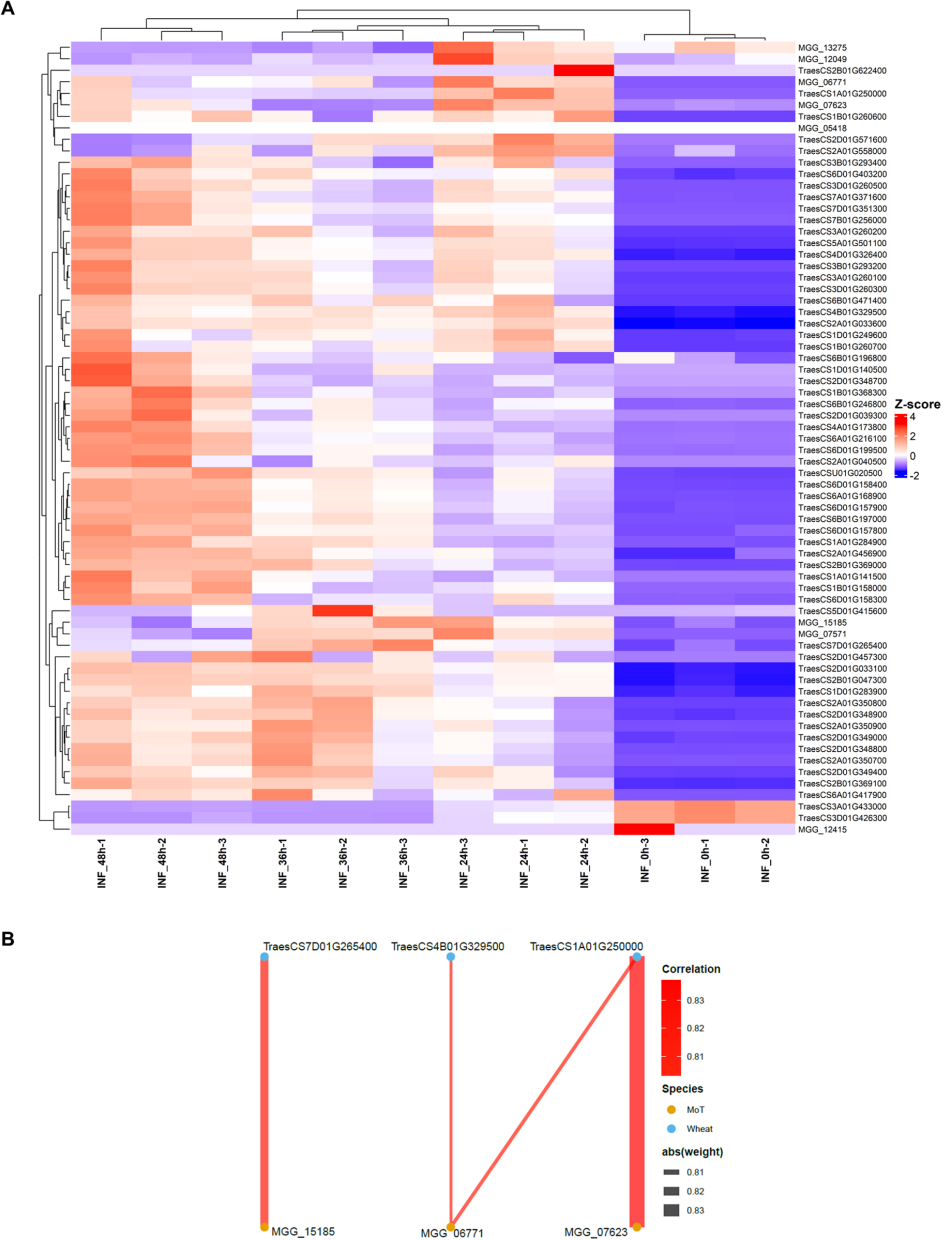
Co-expression analysis of MoT effector candidates and wheat chitin-binding genes. **(A)** Heatmap of co-expression between MoT effector candidates and wheat genes enriched for GO:0008061 (chitin binding) across infection stages. Rows represent genes, columns represent infected samples, and gene expression values were Z-score normalized. Red indicates higher, blue indicates lower, and white represents mean expression. **(B)** Bipartite co-expression network illustrating significant positive correlations (Pearson’s r > 0.8) between MoT effectors (orange nodes) and wheat chitin-binding genes (blue nodes). Edge width is proportional to correlation strength. Exact correlation coefficients are provided in **Supplementary Table 5**.

Notably, the GO term GO:0008061 (chitin binding) was significantly enriched in both MoT effector candidates and wheat genes (**Figures 4A** and **6A**), suggesting that chitin recognition and processing represent key battlegrounds in the host-pathogen interaction. To explore coordinated transcriptional responses between MoT and wheat during infection, we performed comparative expression analysis of chitin-binding genes from both organisms across the infection time course. The heatmap, showing Z-score normalized expression values of chitin-binding genes from both MoT and wheat across four time points (**Figure 8A**), revealed distinct expression patterns between host and pathogen. Pearson correlation (r ≥ 0.8) between MoT chitin-related effector candidates and wheat defense genes was calculated across all four time points, revealing high-confidence co-expression relationships suitable for bipartite network visualization. Four pairs of highly correlated genes were identified, with Pearson correlation coefficients (r) greater than 0.8, indicating strong co-expression relationships between specific MoT effector candidates and wheat genes (**Figure 8B**; **Supplementary Table 5**). The high-correlation pairs are as follows: MGG_15185 and TraesCS7D01G265400 (chitin elicitor receptor kinase 1, CERK1), with a correlation of 0.82; MGG_06771 and TraesCS1A01G250000 (chitinase 2-like, Cht-2), with a correlation of 0.81; MGG_07623 and TraesCS1A01G250000 (Cht-2), with a correlation of 0.84; and MGG_06771 and TraesCS4B01G329500 (chitin elicitor-binding protein-like, CEBiP), with a correlation of 0.80. These co-expression patterns suggest a potential association between the expression of specific MoT effector candidates and wheat genes involved in chitin perception and degradation. Whether these correlations reflect direct molecular interactions or indirect, pathway-level transcriptional coordination remains to be determined. These gene pairs represent high-priority candidates for functional validation to elucidate the molecular mechanisms underlying MoT manipulation of wheat chitin-based immunity.

## 4 Discussion

Compared to the rice-infecting lineage MoO, the wheat-infecting lineage MoT exhibits a higher rate of gene loss and faster evolutionary adaptation, which complicates disease management (Joubert and Krasileva, 2024). Despite the severe impact of wheat blast on global wheat production, the molecular mechanisms underlying MoT infection remain incompletely understood, particularly with respect to how the pathogen establishes infection and how host responses evolve over time. Time-resolved simultaneous-transcriptome analysis of wheat leaves infected with MoT at 0, 24, 36, and 48 hpi revealed pronounced, stage-specific transcriptional reprogramming in wheat, with differential gene expression peaking at 36 hpi and visible disease symptoms emerging by 48 hpi. The 24 hpi transcriptome was characterized by activation of immune- and defense-related processes concurrent with repression of cell wall- and membrane-associated biosynthetic pathways, whereas later stages were marked by pronounced suppression of chloroplast-associated metabolic processes accompanied by sustained activation of immune- and detoxification-related responses. These patterns indicate that MoT infection is accompanied by temporally coordinated remodeling of host defense and metabolic programs. Sustained defense activation coincides with progressive disruption of core metabolic and physiological processes, potentially coinciding with a physiological context permissive to continued pathogen proliferation. Similar temporal decoupling between sustained immune activation and progressive metabolic repression has been described in several compatible plant-hemibiotroph systems (Münch et al., 2008; Zhou et al., 2023). These interpretations are based on transcriptional patterns rather than direct physiological measurements. Causality cannot be inferred from transcriptional data alone.

### 4.1 Early activation of immune responses accompanied by repression of structural biosynthetic processes during early infection

During the initial stages of MoT infection, wheat quickly mobilized innate defense pathways, as evidenced by the enrichment of upregulated genes in response to chitin, defense responses, response to wounding, flavonoid biosynthesis, and glutathione-mediated detoxification. The activation of MAPK signaling and phenylpropanoid biosynthesis is in line with early PAMP-triggered immunity (PTI) signaling (Jones and Dangl, 2006; Bigeard et al., 2015). Homologs annotated to the MPK3/6-associated MAPK (log_2_ Fold Change 1.27–1.68; **Supplementary Table 2**) signaling node in KEGG were transcriptionally upregulated. Meanwhile, the enrichment of cytochrome P450–mediated xenobiotic metabolism and glutathione metabolism suggests rapid engagement of detoxification and redox homeostasis mechanisms in response to infection-associated stress (Chakraborty et al., 2023). For example, glutathione transferases Alpha 1 (GSTA1), encode Phi GST enzymes and helps defend against stress, were strongly induced (log_2_ fold change 2.41-6.50 across homologs, **Supplementary Table 2**). Collectively, these transcriptional changes indicate that wheat recognizes MoT and mounts a rapid defense-associated responses at 24 hpi.

Concurrently, downregulated genes were enriched in cell wall and membrane biosynthesis as well as cytoskeleton-related processes, including cellulose biosynthesis, phospholipid and choline metabolism, wax and cutin biosynthesis, and microtubule-associated processes. Phytopathogenic microorganisms deploy cell wall–degrading enzymes (CWDEs) as virulence factors, while plants can produce inhibitors of CWDEs; this interaction reduces cell wall damage and promotes the accumulation of cell wall–derived fragments that act as damage-associated molecular patterns (DAMPs), thereby inducing PTI (Lorrai and Ferrari, 2021).

This transcriptional pattern does not indicate a failure of immune activation but instead reflects a coordinated defense-associated transcriptional shift. Such transcriptional changes are consistent with early immune-associated remodeling of structural components and a shift in transcriptional priorities toward defense-related processes (Wan et al., 2021; He et al., 2022b). The biosynthesis of cell wall components and lipid membranes is energetically demanding, and its transient downregulation is a pattern previously reported in a transcriptional shift away from growth- and structure-associated biosynthesis toward defense-associated pathways (Rojas et al., 2014). Such resource reprioritization is a common strategy in plants during stress responses (Ku et al., 2022), enabling the rapid deployment of immune signaling and secondary metabolism (e.g., flavonoid and phenylpropanoid pathways) at the expense of structural biosynthesis (Huot et al., 2014).

Taken together, the 24 hpi transcriptional landscape indicates that wheat rapidly activates immune and detoxification programs while transiently downregulating growth- and structure-related biosynthetic pathways. This coordinated response delineates 24 hpi as a rapid activation phase, setting the stage for subsequent metabolic reprogramming and physiological adjustments as infection progresses.

### 4.2 Sustained immune activation accompanied by systemic repression of chloroplast-associated functions

At 36 hpi, wheat transcriptional responses were characterized by the sustained activation of immune- and detoxification-related pathways, concomitant with a coordinated and systemic repression of chloroplast-associated and photosynthetic processes. Functional enrichment analyses revealed continued upregulation of canonical defense-related pathways, including response to chitin, defense responses, phenylpropanoid biosynthesis, glutathione metabolism, and MAPK signaling, indicating that host immune activation initiated at 24 hpi is maintained during mid-infection. Enrichment of alpha-linolenic acid metabolism is consistent with activation of lipid-derived signaling pathways, potentially associated with jasmonate-related defense responses (Zhu et al., 2024). PAD4 homologs showed consistent transcriptional elevation (log_2_ fold change 1.74–1.99 across PAD4 homologs, **Supplementary Table 2**), while EDS1 exhibited moderate induction below the DEG threshold, together suggesting engagement of EDS1-PAD4-associated signaling, accompanied by enrichment of hypersensitive response–related GO terms (p = 3.45 × 10^−7^). These patterns may be consistent with the emergence of ETI-associated transcriptional signatures during mid-infection, while PTI-related pathways remain dominant.

In contrast, a coordinated downregulation of chloroplast- and photosynthesis-related functions was observed at 36 hpi. Downregulated genes were strongly enriched in light-harvesting complexes, photosystem I and II components, chloroplast thylakoid membranes, pigment binding, and carbon fixation pathways. Notably, light-harvesting chlorophyll (a/b)-binding (LHCA and LHCB) protein superfamily were broadly downregulated (**Supplementary Table 2**), responsible for capturing light energy and transferring it to photosystems to drive photosynthesis. LHCA associated with Photosystem I (PSI) and LHCB associated with Photosystem II (PSII) (Damkjær et al., 2009), further supporting the coordinated transcriptional repression of chloroplast-associated functions. This coordinated repression of chloroplast-associated function is consistent with a systemic downscaling of photosynthetic capacity rather than stochastic gene-level variation (Caffarri et al., 2014). Similar transcriptional suppression of chloroplast-associated processes has been reported in multiple compatible interactions involving hemibiotrophic pathogens and has been associated with altered metabolic prioritization, altered chloroplast-associated immune signaling, and the gradual establishment of a metabolic environment commonly reported in susceptible host-pathogen systems (Liu et al., 2024; Cheaib and Killiny, 2025; Rui et al., 2025). Whether transcriptional suppression of chloroplast immunity represents a common strategy in compatible interactions across diverse pathosystems remains to be determined.

Chloroplast immunity has emerged as a cornerstone of plant defense and a target of plant pathogens, integrating environmental sensing, immune signal generation, inter-organellar communication, and dynamic structural remodeling to orchestrate robust PTI- and ETI-associated responses (Liu et al., 2024). Consistently, chloroplasts are frequent targets of pathogen effector-mediated immune suppression. Diverse pathogens secrete effectors that directly or indirectly impair chloroplast-associated immune functions, including the *Sclerotinia sclerotiorum* effector SsCm1 (Xiao et al., 2025), the *Ralstonia solanacearum* Type III Effector RipAL (Nakano and Mukaihara, 2018), and *Pseudomonas syringae* effector HopN1 (Rodríguez-Herva et al., 2012). Although MoT effector candidates identified in this study were not annotated with chloroplast-targeting functions, the possibility that MoT interferes with chloroplast-mediated immunity through uncharacterized mechanisms warrants further investigation.

Together, the transcriptional profile represents a transitional phase where wheat maintains active defense and detoxification responses while photosynthesis- and chloroplast-associated functions are broadly downregulated, consistent with a systemic reprogramming of host metabolism to prioritize defense over growth, preceding visible lesions.

### 4.3 Escalation of defense-associated metabolic constraints during lesion development

While transcriptional repression of chloroplast-associated functions becomes apparent at 36 hpi, the 48 hpi transcriptome reflects a qualitatively different state marked by extensive repression of primary metabolism coinciding with lesion initiation. Wheat maintains metabolically costly immune- and detoxification-related responses, while extensive repression of photosynthesis, chloroplast maintenance, and primary metabolism indicates substantial metabolic strain and progressive impairment of metabolic homeostasis.

Consistent with the transcriptional patterns observed at 36 hpi, components of the EDS1-PAD4 signaling module remained transcriptionally elevated at 48 hpi (**Supplementary Table 2**). The sustained expression of these ETI-associated components coincides with lesion initiation and extensive metabolic repression, suggesting continued engagement of EDS1-PAD4-associated immune signaling under conditions of increasing physiological stress. The pattern mirrors transcriptional trends reported for hemibiotrophic pathogens that exhibit stage-dependent extensive repression of photosynthesis to prioritize defense, such as *Colletotrichum lindemuthianum* infection (Meyer et al., 2001; Oblessuc et al., 2012). The enrichment of phenylalanine ammonia-lyase activity, glutathione transferase activity, and chitinase activity supports continued engagement of enzymatic defense machinery during lesion formation (van Loon et al., 2006).

In addition to canonical defense-related terms, several enriched categories at 48 hpi reflected sustained stress-associated responses rather than pathogen-specific immune programs, consistent with a prolonged, stress-responsive defense signature under sustained stress conditions (Song et al., 2021). The emergence of nutrient reservoir activity (*p* = 2.13×10^-16^) reflects significant transcriptional enrichment of genes annotated with nutrient reservoir activity, suggesting altered regulation of internal resource allocation under severe stress, a feature frequently associated with late-stage infection or prolonged stress, suggesting altered reliance on endogenous resources to support ongoing metabolic demands (Häffner et al., 2015). Together with the coordinated repression of photosynthetic machinery and pathways implicated in chloroplast maintenance and metabolic homeostasis, these patterns suggest that chloroplast-associated function may be compromised beyond energy production, potentially affecting broader aspects of organellar regulation (Sun et al., 2025).

In line with this interpretation, repression of chloroplast- and photosynthesis-associated functions became more extensive and pronounced at 48 hpi. Genes involved in light harvesting, photosystem I and II components, carbon fixation, chlorophyll and carotenoid biosynthesis, photorespiration, and central carbon metabolism were consistently downregulated. For example, LHCA and LHCB remained transcriptionally downregulated (**Supplementary Table 2**). This widespread transcriptional repression, together with reduced expression of pathways linked to starch, sucrose, and pentose phosphate metabolism, suggests a pronounced reduction in primary metabolic activity rather than a finely regulated adjustment (Berger et al., 2007; Stincone et al., 2015; Wu et al., 2024). Such widespread repression in line with increasing metabolic constraints accompanying tissue damage and symptom development. This pattern aligns with the characteristic progression of hemibiotrophic pathogens, in which the early biotrophic phase maintains metabolically active host tissue, whereas later necrotrophic phases coincide with host cell death and tissue compromise (Münch et al., 2008; Gan et al., 2013). Within this framework, extensive suppression of photosynthesis and primary metabolism is commonly observed during compatible interactions as host tissues lose metabolic integrity during lesion expansion.

Despite sustained activation of immune and detoxification pathways, disease symptoms progressed, suggesting that prolonged defense engagement may impose significant metabolic demand, potentially overwhelming the host’s energy and redox capacity during lesion formation. In the hemibiotrophic pathogen *Phytophthora infestans*, a hypothetical model has been proposed in which effectors expressed during early biotrophic stages suppress programmed cell death (PCD), whereas effectors induced at later stages promote rapid cell death and tissue necrosis following extensive pathogen proliferation (Lee and Rose, 2010). Further investigation is needed to elucidate the functional role of effector candidates in inducing PCD.

Taken together, this transcriptional configuration suggests that continued defense engagement at this stage imposes metabolic constraints, rather than reflecting effective immune containment of the pathogen. At 48 hpi, wheat transcriptomes define a defense and metabolism imbalance, in which sustained and metabolically costly immune and xenobiotic responses coincide with extensive repression of photosynthesis, chloroplast maintenance, and primary carbon metabolism, consistent with a marked impairment of metabolic homeostasis during lesion initiation.

### 4.4 Effector candidates and their potential impact on host processes

Our transcriptome-based analysis identified multiple upregulated MoT effector candidates, including both well-characterized orthologs of MoO and additional proteins with features typical of fungal effectors. Many of the known effectors exhibit functions related to immune suppression, regulation of programmed cell death, or manipulation of host metabolism. For instance, MoHrip1 (MGG_15022), an Alt a 1 family of proteins (AA1s), binds to the plant plasma membrane and induces ROS accumulation, callose deposition, alkalization and necrosis (Chen et al., 2012; Zhang et al., 2017), while MoHrip2 (MGG_16187) enhances virulence by targeting the apoplastic osmotin-like protein OsOLP1 (Yan et al., 2025). Cell death-inducing proteins such as MoCDIP1 (MGG_03356) were upregulated during infection (Wei et al., 2023), whereas MoCDIP6 (MGG_01532), although capable of triggering apoptosis, has been reported to be dispensable for pathogenicity (Guo et al., 2019). Nup1 (MGG_07900), a component of the nuclear pore complex, suppresses BAX-triggered PCD in *N. benthamiana* (Dong et al., 2015). SPD5 (MGG_02154), a homologue of BAS4, delineates hyphae during the biotrophic phase, whereas SPD11 (MGG_05344), upregulated at 48 hpi, is a homolog of *Colletotrichum higginsianum* effector ChEC5 and required for *M. oryzae* virulence (Sharpee et al., 2017). Except for SPD11, most of these showed high expression starting at 24 hpi, consistent with roles in association with early immune responses, whereas SPD11 peaks at 48 hpi, coinciding with lesion initiation.

A second group of effectors was strongly expressed at 36 hpi. Pwl2 is encoded by two identified loci, MGG_13863 and MGG_04301 (Were et al., 2025). In rice, Pwl2 has been characterized as a G-type lectin receptor-like kinase that negatively regulates resistance by maintaining ROS homeostasis and modulating PCD (Xu et al., 2023). In barley, Pwl2 is recognized by the Mla3 immune receptor, targets HIPP43 from plasmodesmata and promotes rapid tissue colonization (Brabham et al., 2024; Were et al., 2025). *Avr*-Pi54 (MGG_03685) functions in the rice nucleus as either a transcription factor or chromatin remodeler, contributing to host immunocompromization (Saklani et al., 2023). The hydrophobin MPG1 (MGG_10315) integrates signals from multiple upstream pathways, modulating MAPK, cAMP responses, and nitrogen repression pathways to facilitate host invasion (Soanes et al., 2002; Wei et al., 2023). The temporal expression of these effectors aligns with the onset of metabolic reprogramming in wheat, suggesting functional roles during the mid-to-late infection phase.

Beyond known MoO orthologs, several additional effector-like proteins were identified. These include Alt a protein family, a unique group of fungal secreted proteins with β-barrel dimers and major allergen produced by *Alternaria alternata* (Chruszcz et al., 2012; Qiu et al., 2024). CV-N homologs, which bind carbohydrates to inhibit viral fusion (Xavier et al., 2024). Metalloproteinases, including Zinc metalloproteinase, which degrade host tissues and suppress immunity (Wang et al., 2024). One putative MoT metalloproteinase (MGG_05515), containing a metallopeptidase catalytic domain, was strongly upregulated and may target wheat zinc-binding protein to promote virulence (Cerdà-Costa and Gomis-Rüth, 2014; Wang et al., 2024). Collectively, these effectors represent a multifaceted toolkit potentially acting at different stages to influence host immunity, rewire metabolism, and regulate host cell death.

### 4.5 Effector candidates may modulate chitin-triggered immunity in wheat

Chitin binding is a central event in plant and fungal interactions, enabling the host to detect invading fungi while also providing pathogens with potential targets for immune modulation. In rice, suppression of chitin-triggered immunity is essential for rice blast development (Mentlak et al., 2012). Whether MoT employs comparable strategies in wheat remains to be experimentally determined.

In this study, chitin-associated GO terms were enriched in both wheat and MoT transcriptomes, suggesting that the chitin receptor interface may represent a major site of host-pathogen interaction. Co-expression analysis revealed a strong correlation between chitin elicitor receptor kinase 1 (CERK1) and chitin elicitor-binding protein (CEBiP), both key sensors for chitin perception and LysM proteins. CERK1 perceives chitooligosaccharides and activates downstream immune signaling, while two CEBiP molecules simultaneously bind to a single N-acetylchitoheptaose/octaose through their central LysM domain and dimerize (Hayafune et al., 2014). The ligand dependent CEBiP-CEBiP homodimers form a chitin receptor complex, and both TaCERK1 and TaCEBiP contribute to pathogen *Mycosphaerella graminicola* virulence (Lee et al., 2014).

In rice, the LysM effector Slp1 competes with CEBiP for chitin oligosaccharide binding, thereby facilitating tissue invasion and lesion expansion (Mentlak et al., 2012). In our database, the MoO LysM protein Slp2 (MGG_03468), which is not expressed during infection in rice (Mentlak et al., 2012), showed transient induction at 24 hpi during MoT infection, but no sustained significant upregulation thereafter. Another LysM-containing candidate, MGG_07571, was significantly induced across 24, 36 and 48 hpi. Although LysM domain enrichment was not statistically significant, the observed expression patterns suggest that these candidates may participate in modulating chitin-triggered immunity, either directly or indirectly.

A wheat chitinase gene, Cht-2, showed strong co-expression with effector candidates. Cht-2 encodes a chitinase capable of degrading fungal cell walls and enhanced resistance to *Magnaporthe grisea* when overexpressed in rice (Nishizawa et al., 1999). Co-expression patterns observed here indicate a potential interaction between MoT effectors and wheat chitinase-mediated defenses, highlighting priority candidates for experimental validation.

These candidate pairs may represent direct or indirect modulation of chitin-triggered immunity and thus define high-priority targets for future experimental validation in wheat. KEGG analysis confirmed sustained activation of defense and detoxification pathways. Together, these results support a temporally structured model in which sustained immune activation coincides with progressive repression of photosynthesis-associated processes and stage-specific effector deployment, highlighting chitin-related host-pathogen interfaces as high-priority targets for experimental validation.

### 4.6 Limitations and future direction

Based on these findings, we propose a three-phase transcriptional model associated with wheat susceptibility in which MoT infection triggers rapid immune activation that becomes progressively metabolically constraining and ultimately insufficient to prevent disease progression. Several limitations warrant consideration. Although the B71 genome offers lineage-specific context, its incomplete annotation limits precise interpretation at the effector level. Reliance on a single MoT isolate and detached-leaf inoculation may not fully represent effector expression under natural, tissue-diverse, or genotype-diverse conditions. Transcriptome data alone cannot capture spatial heterogeneity or post-transcriptional and protein-level regulation. Accordingly, our study focuses on temporal dynamics of fungal gene expression and host transcriptional reprogramming, rather than providing a comprehensive catalog of MoT effectors. RNA-seq results were derived from three independent biological replicates per time point, and temporal transcriptional patterns are consistent across replicates, supporting the reliability of observed DEG trends. Although independent experimental validation (e.g., via qRT-PCR) was not feasible under current biosafety restrictions, this remains an important direction. The effector candidates identified here highlight potential mechanisms by which MoT may interfere with host immune perception, modulate defense signaling, and influence broader stress-associated transcriptional responses. Future work will require systematic functional validation, including molecular cloning, interaction screening (e.g., yeast two-hybrid assays), and biochemical approaches (e.g., pull-down and co-immunoprecipitation).

## 5 Conclusion

This study delineates the temporal organization of wheat transcriptional responses during the early stages of wheat blast infection through integrated host-pathogen transcriptomes. The data reveal a sequential progression of host states. At 24 hpi, wheat exhibits rapid transcriptional activation of innate immunity and secondary metabolic pathways, consistent with early pathogen recognition. By 36 hpi, immune-associated transcription remains elevated, while photosynthesis- and chloroplast-related functions are broadly reduced, indicating a shift in resource allocation. At 48 hpi, transcriptomic profiles reflect a pre-necrotic condition marked by continued defense-associated transcription together with widespread attenuation of primary metabolic processes. These phases likely represent a continuum rather than sharply discrete transitions.

Collectively, these observations support a model in which sustained defense investment coincides with increasing metabolic constraint during wheat disease development. By resolving these stage-dependent transcriptional transitions, this work provides a clearer framework for interpreting host responses during the initial phases of wheat blast. These findings provide a transcriptome-informed context for interpreting host responses and may help guide future efforts to improve wheat blast resistance, informing future transcriptome-guided breeding or molecular screening efforts.

## Data Availability

The raw reads of sequencing data have been deposited in the NCBI Bio Project database under accession number PRJNA1392009.

## Supplementary material

Supplementary Figure 1 Phenotypic appearance of mock-inoculated wheat seedlings. Leaves of mock-inoculated (CK) seedlings at 0, 24, 36, and 48 hpi. Leaves were treated with 0.25% gelatin solution and showed no visible disease symptoms throughout the experimental period.

Supplementary Figure 2 Baseline transcriptional variation at 0 hpi. Volcano plot showing DEGs between mock-inoculated and MoT-inoculated samples at 0 hpi, representing baseline transcriptional differences prior to infection. Upregulated genes are shown in red and downregulated genes in blue.

Supplementary Figure 3 Overlap of total DEGs across infection stages. Venn diagram showing shared and stage-specific differentially expressed genes (DEGs) identified at 24, 36, and 48 hpi between mock-inoculated and MoT-infected wheat leaves. Circles represent DEGs detected at each time point, colored yellow (24 hpi), orange (36 hpi), and green (48 hpi).

Supplementary Figure 4 Hierarchical clustering of infection samples mapped to MoT. Hierarchical clustering based on variance-stabilized RNA-seq counts shows tight grouping of biological replicates within each time point, consistent with the temporal progression of infection.

Supplementary Data 1 Sample metadata Table lists all samples used in the study, including organism, condition (infected or mock), time point post infection, and biological replicate. Sample IDs correspond to column headers in **Supplementary Datas 2**–**3** count matrices.

Supplementary Data 2 Wheat raw count matrix Gene-level raw read counts for all wheat samples. Columns correspond to Sample IDs in **Supplementary Data 1**. Counts are integers and were used for differential expression analysis.

Supplementary Data 3 MoT raw count matrix Gene-level raw read counts for Magnaporthe oryzae Triticum (MoT). Only infected wheat leaf samples are included, as mock-inoculated controls do not contain MoT reads. Column headers correspond to Sample IDs listed in **Supplementary Data 1**.

Supplementary Table 1 Comprehensive annotation of predicted MoT effector candidates. This table summarizes all predicted MoT effector candidates identified in this study. For each candidate, information is provided on effector localization prediction (cytoplasmic or apoplastic), signal peptide presence and cleavage site, transmembrane domains, GPI-anchor prediction, subcellular localization, protein length, molecular weight, cysteine content, conserved motifs, PFAM annotations, and sequence similarity to UniRef90 proteins. These annotations were used to support effector candidate identification and downstream functional analyses. An additional worksheet summarizes domain enrichment results for MoT effector candidates based on DAVID analysis, integrating annotations from the InterPro, Pfam, and SMART databases. Enriched domains include chitin-binding, endochitinase-like, metallopeptidase-associated, and DUF-containing families, with associated statistical parameters and contributing genes. This domain-level annotation provides complementary insight into conserved structural features and potential functional properties of MoT effector candidates.

Supplementary Table 2 DEGs in wheat during MoT infection. This table lists wheat genes showing differential expression between MoT-infected and mock-treated samples at 0, 24, 36, and 48 hpi. Separate worksheets correspond to individual time-point comparisons. For each gene, normalized expression values, log_2_ fold changes, statistical significance, regulation trends, and functional annotations are provided. DEGs detected at 0 hpi likely reflect baseline variation associated with inoculation or early handling and are included for completeness; biological interpretation in the manuscript focuses on responses from 24 hpi onward.

Supplementary Table 3 GO enrichment analysis of wheat and MoT genes across infection stages. This table presents Gene Ontology enrichment results for wheat differentially expressed genes at 24, 36, and 48 hpi. For each time point, upregulated and downregulated genes (infected vs. mock) were analyzed separately and are provided in distinct worksheets. Enriched biological processes, cellular components, and molecular functions are reported with associated statistics and gene lists. The final worksheet summarizes GO enrichment results for MoT genes, providing a global view of pathogen functional dynamics during infection.

Supplementary Table 4 KEGG pathway enrichment of wheat transcriptional responses to MoT infection. This table summarizes KEGG pathway enrichment analyses for wheat differentially expressed genes at 24, 36, and 48 hpi. Upregulated and downregulated gene sets (infected vs. mock) were analyzed separately for each time point and are presented in individual worksheets. Enriched pathways are annotated by functional classification, statistical significance, and contributing genes, providing insight into stage-specific metabolic and signaling pathways associated with wheat blast infection.

Supplementary Table 5 Expression correlations between MoT effector candidates and wheat genes. This table shows MoT effector candidates whose expression levels are strongly correlated with those of wheat genes across infection time points. Pearson correlation coefficients are reported for each effector–host gene pair, suggesting potential links between pathogen effector activity and host transcriptional responses during wheat blast infection.
