# Supplementary material for: Transcriptomic profiling of wheat (Triticum Aestivum L.) response to infection by the wheat blast fungus Magnaporthe Oryzae Triticum
Source: Front Plant Sci. 2026 Mar 5;17:1776686. doi: 10.3389/fpls.2026.1776686 (PMC12999787; doi:10.3389/fpls.2026.1776686)
Supplement: Supplementary Figure 1 — Phenotypic appearance of mock-inoculated wheat seedlings. Leaves of mock-inoculated (CK) seedlings at 0, 24, 36, and 48 hpi. Leaves were treated with 0.25% gelatin solution and showed no visible disease symptoms throughout the experimental period. [file DataSheet4.docx]

Supplementary Material

# Supplementary Figures and Tables

## Supplementary Figures

**Supplementary Figure 1. Phenotypic appearance of mock-inoculated wheat seedlings.**

Leaves of mock-inoculated (CK) seedlings at 0, 24, 36, and 48 hpi. Leaves were treated with 0.25% gelatin solution and showed no visible disease symptoms throughout the experimental period.


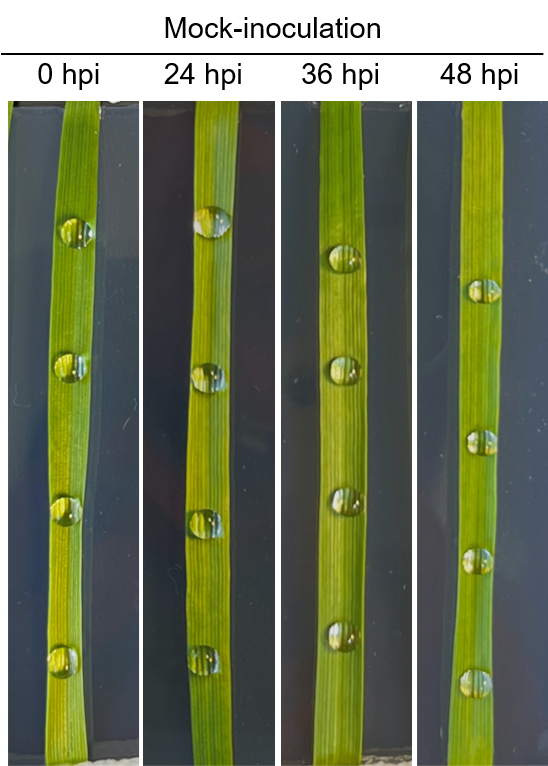


**Supplementary Figure 2. Baseline transcriptional variation at 0 hpi.**

Volcano plot showing DEGs between mock-inoculated and MoT-inoculated samples at 0 hpi, representing baseline transcriptional differences prior to infection. Upregulated genes are shown in red and downregulated genes in blue.


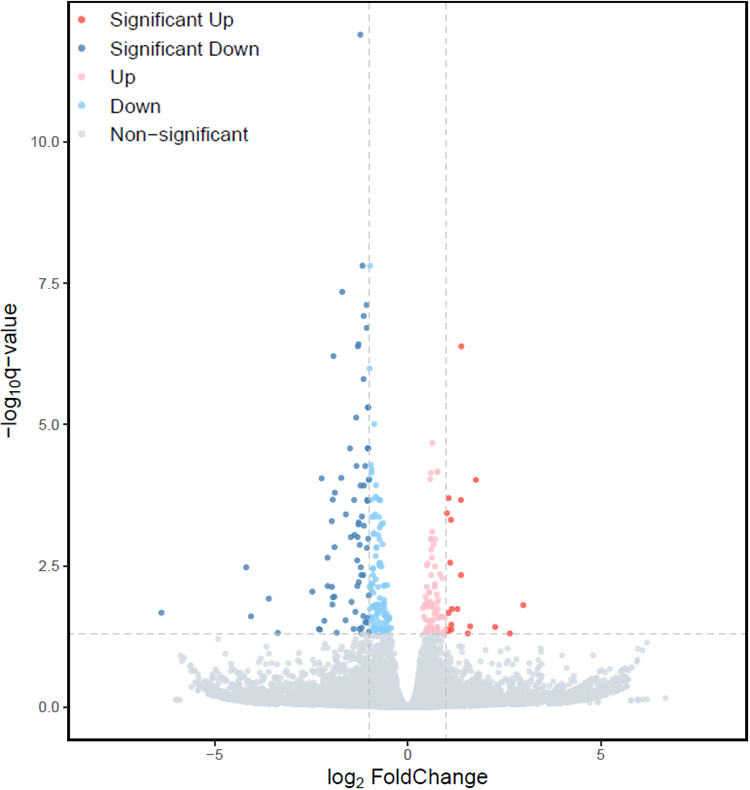


**Supplementary Figure 3. Overlap of total DEGs across infection stages.**

Venn diagram showing shared and stage-specific differentially expressed genes (DEGs) identified at 24, 36, and 48 hpi between mock-inoculated and MoT-infected wheat leaves. Circles represent DEGs detected at each time point, colored yellow (24 hpi), orange (36 hpi), and green (48 hpi).
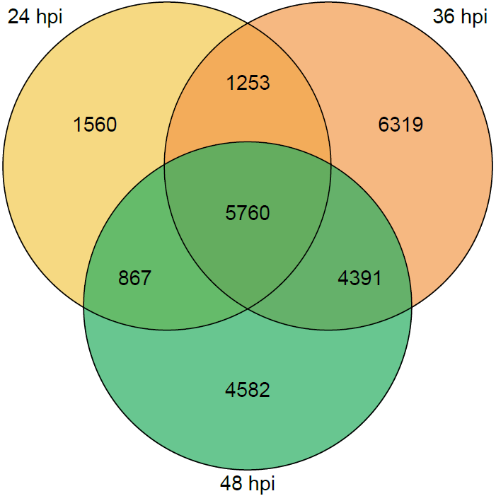


**Supplementary Figure 4. Hierarchical clustering of infection samples mapped to MoT.**

Hierarchical clustering based on variance-stabilized RNA-seq counts shows tight grouping of biological replicates within each time point, consistent with the temporal progression of infection.
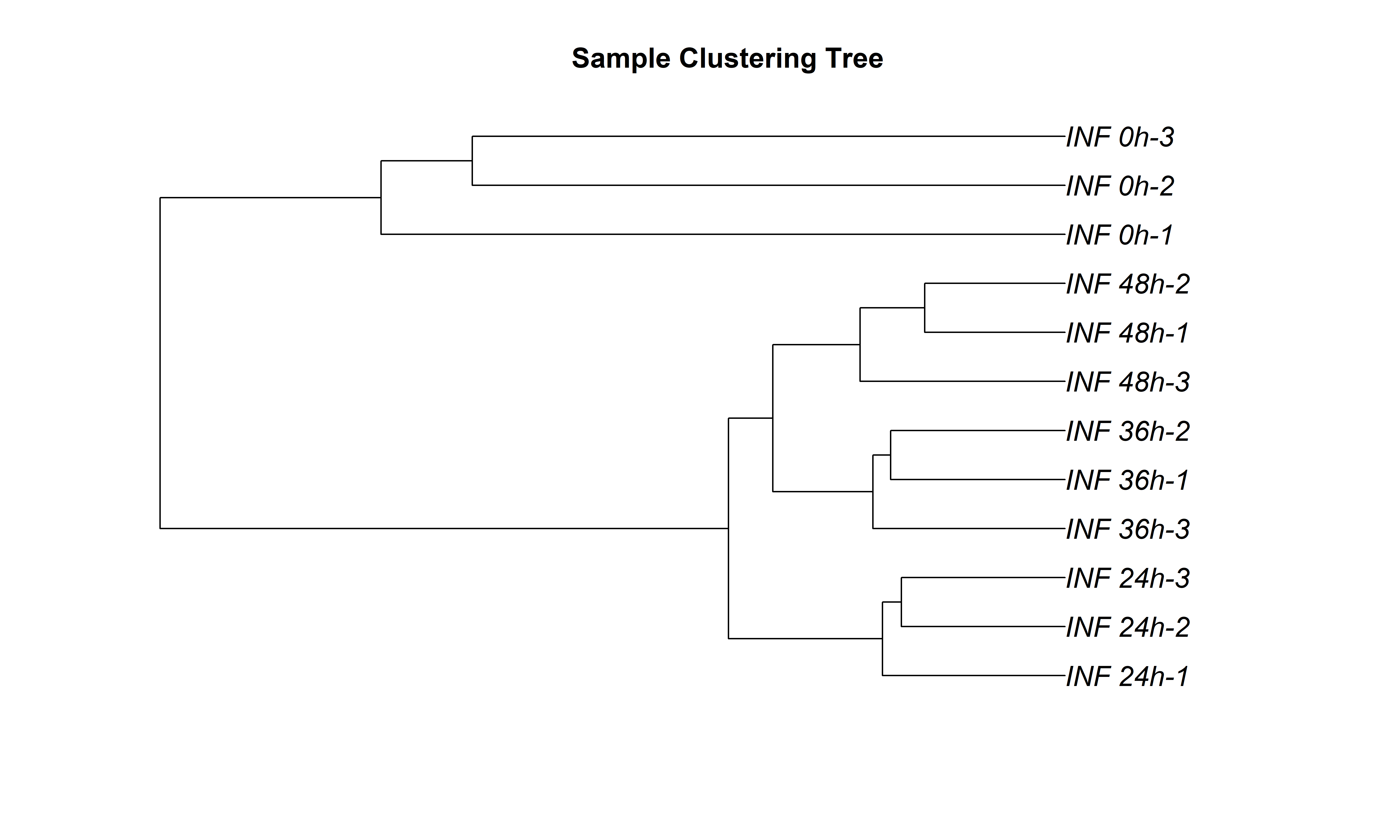


## Supplementary Tables

**Supplementary Table 1. Comprehensive annotation of predicted MoT effector candidates.**

This table summarizes all predicted MoT effector candidates identified in this study. For each candidate, information is provided on effector localization prediction (cytoplasmic or apoplastic), signal peptide presence and cleavage site, transmembrane domains, GPI-anchor prediction, subcellular localization, protein length, molecular weight, cysteine content, conserved motifs, PFAM annotations, and sequence similarity to UniRef90 proteins. These annotations were used to support effector candidate identification and downstream functional analyses.

An additional worksheet summarizes domain enrichment results for MoT effector candidates based on DAVID analysis, integrating annotations from the InterPro, Pfam, and SMART databases. Enriched domains include chitin-binding, endochitinase-like, metallopeptidase-associated, and DUF-containing families, with associated statistical parameters and contributing genes. This domain-level annotation provides complementary insight into conserved structural features and potential functional properties of MoT effector candidates.

**Supplementary Table 2. DEGs in wheat during MoT infection.**

This table lists wheat genes showing differential expression between MoT-infected and mock-treated samples at 0, 24, 36, and 48 hpi. Separate worksheets correspond to individual time-point comparisons. For each gene, normalized expression values, log_2_ fold changes, statistical significance, regulation trends, and functional annotations are provided. DEGs detected at 0 hpi likely reflect baseline variation associated with inoculation or early handling and are included for completeness; biological interpretation in the manuscript focuses on responses from 24 hpi onward.

**Supplementary Table 3. GO enrichment analysis of wheat and MoT genes across infection stages.**

This table presents Gene Ontology enrichment results for wheat differentially expressed genes at 24, 36, and 48 hpi. For each time point, upregulated and downregulated genes (infected vs. mock) were analyzed separately and are provided in distinct worksheets. Enriched biological processes, cellular components, and molecular functions are reported with associated statistics and gene lists. The final worksheet summarizes GO enrichment results for MoT genes, providing a global view of pathogen functional dynamics during infection.

**Supplementary Table 4. KEGG pathway enrichment of wheat transcriptional responses to MoT infection.**

This table summarizes KEGG pathway enrichment analyses for wheat differentially expressed genes at 24, 36, and 48 hpi. Upregulated and downregulated gene sets (infected vs. mock) were analyzed separately for each time point and are presented in individual worksheets. Enriched pathways are annotated by functional classification, statistical significance, and contributing genes, providing insight into stage-specific metabolic and signaling pathways associated with wheat blast infection.

**Supplementary Table 5. Expression correlations between MoT effector candidates and wheat genes.**

This table shows MoT effector candidates whose expression levels are strongly correlated with those of wheat genes across infection time points. Pearson correlation coefficients are reported for each effector-host gene pair, suggesting potential links between pathogen effector activity and host transcriptional responses during wheat blast infection.

## Supplementary Data

**Supplementary Data 1 Sample metadata**

Table lists all samples used in the study, including organism, condition (infected or mock), time point post infection, and biological replicate. Sample IDs correspond to column headers in Supplementary Data 2–3 count matrices.

**Supplementary Data 2 Wheat raw count matrix**

Gene-level raw read counts for all wheat samples. Columns correspond to Sample IDs in Supplementary Data 1. Counts are integers and were used for differential expression analysis.

**Supplementary Data 3 MoT raw count matrix**

Gene-level raw read counts for *Magnaporthe oryzae* *Triticum* (MoT). Only infected wheat leaf samples are included, as mock-inoculated controls do not contain MoT reads. Column headers correspond to Sample IDs listed in Supplementary Data 1.
